# Balancing lipid synthesis and oxidation promotes B cell response to vaccination during immunosuppression

**DOI:** 10.1172/JCI205170

**Published:** 2026-09-17

**Authors:** Elizabeth A. Thompson, Alexis Figueroa, Katerina Roznik, Nicole E. Skinner, Santosh Dhakal, Shuai Li, Luca Biavati, Laura A. Sena, Laila Stoddart, Karli Redinger, Samuel B. Warner, Sabra L. Klein, Nadine Rouphael, Joel N. Blankson, Yolanda Eby, Robert D. Leone, Peter S. Heeger, Mark A. Robien, Christian P. Larsen, Erika L. Pearce, Edward J. Pearce, Hongkai Ji, Andrew H. Karaba, Dorry L. Segev, Aaron A.R. Tobian, William A. Werbel, Andrea L. Cox, Justin R. Bailey

**Affiliations:** 1 Department of Medicine, Johns Hopkins University School of Medicine, Baltimore, Maryland, USA.; 2 Department of Molecular Microbiology and Immunology, Johns Hopkins University Bloomberg School of Public Health, Baltimore, Maryland, USA.; 3 Division of Infectious Diseases, Department of Medicine, The Ohio State University Wexner Medical Center, Columbus, Ohio, USA.; 4 Center for Vaccines and Immunity, The Abigail Wexner Research Institute, Nationwide Children’s Hospital, Columbus, Ohio, USA.; 5 Department of Diagnostic Medicine/Pathobiology, College of Veterinary Medicine, Kansas State University, Manhattan, Kansas, USA.; 6 Johns Hopkins Bloomberg School of Public Health, Department of Biostatistics, Baltimore, Maryland, USA.; 7 Department of Oncology, Johns Hopkins University School of Medicine, Baltimore, Maryland, USA.; 8 Hope Clinic, Infectious Diseases Division, Emory University, Decatur, Georgia, USA.; 9 Department of Pathology, Johns Hopkins University School of Medicine, Baltimore, Maryland, USA.; 10 Department of Medicine, Cedars-Sinai Medical Center, Los Angeles, California, USA.; 11 Transplantation Branch, Division of Allergy Immunology and Transplantation, National Institute of Allergy and Infectious Diseases (NIAID), NIH, Rockville, Maryland, USA.; 12 Department of Surgery, Emory University, Atlanta, Georgia, USA.; 13 Department of Biochemistry and Molecular Biology, Johns Hopkins University Bloomberg School of Public Health, Baltimore, Maryland, USA.; 14 Department of Surgery, New York University Grossman School of Medicine, New York, New York, USA.

**Keywords:** Immunology, Metabolism, Adaptive immunity, B cells, Fatty acid oxidation

## Abstract

Pharmacologic immunosuppression is essential for preventing organ rejection and controlling autoimmunity but profoundly impairs humoral immunity, increasing the risk of vaccine failure and infection. The mechanisms by which immunosuppressive therapies disrupt human B cell responses remain poorly defined. Here, we identified dysregulated lipid metabolism as a central determinant of impaired vaccine response in solid organ transplant recipients (SOTRs). Using high-dimensional immune profiling, single-cell transcriptomics, and functional metabolic assays, we found that effective B cell responses required a homeostatic balance between lipid synthesis and fatty acid oxidation (FAO). The widely used immunosuppressive agent mycophenolic acid (MPA) was strongly associated with vaccine nonresponses and induced excessive lipid synthesis, lipid accumulation, and mitochondrial stress in B cells. In contrast, CD11c^+^ B cells retained the capacity to differentiate into plasmablasts in the presence of MPA through elevated expression of CPT1a, a mitochondrial FA transporter, and enhanced FAO. These cells were found to be a key feature of early effective vaccine responses in healthy individuals and SOTRs. Notably, pharmacologic inhibition of cholesterol synthesis partially restored plasmablast differentiation in the presence of MPA. These findings identify B cell lipid metabolism as a critical and targetable regulator of human humoral immunity during immunosuppression.

## Abstract 2

This study identifies B cell lipid metabolism as a critical and targetable regulator of human humoral immunity during immunosuppression.

## Graphical abstract

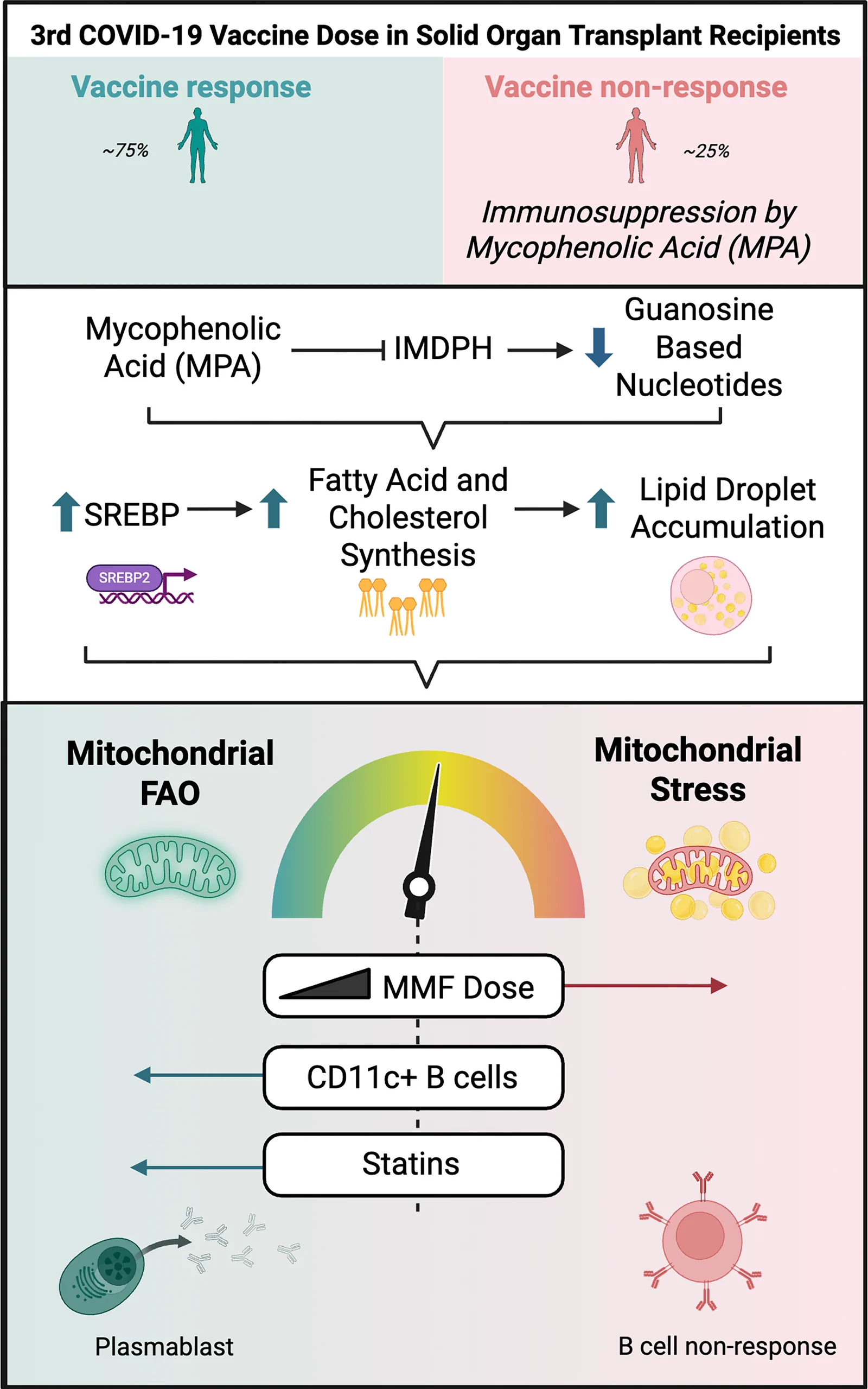

## Introduction

Effective humoral immunity requires the coordinated activation, differentiation, and metabolic adaptation of B cells, yet these processes are profoundly disrupted in immunosuppressed individuals. Immunosuppression is increasingly common, affecting an estimated 6.6% of US adults in 2021 compared with 2.7% in 2013 (1). Solid organ transplant recipients (SOTRs) and patients treated for autoimmune disease mount weaker Ab responses to vaccination and infection than do immunocompetent individuals (2). SOTRs in particular have reduced vaccine-induced seroconversion and Ab production, contributing to severe infections across pathogens (3–6). Thus, strategies are needed to enhance protective immunity without increasing organ rejection or autoimmune flares.

Vaccination provides a controlled and physiologically relevant system to interrogate the determinants of successful human B cell responses under immunosuppression. We and others have shown that additional doses of SARS-CoV-2 vaccine can lead to seroconversion and induction of spike-specific (S-specific) Abs in a subset of vaccinated SOTRs, but a substantial portion remain nonresponsive despite repeated antigen exposure (7–12). The immunological mechanisms underlying this heterogeneity remain poorly defined. Therefore, understanding basic human B cell biology in the context of immunosuppression could facilitate the development of novel strategies to enhance protective immune responses during immunosuppression. Furthermore, evaluating immune responses in SOTRs offers a unique opportunity to identify key determinants of vaccine response in humans by evaluating immune responses when specific immunological or metabolic pathways are inhibited through pharmaceutical interventions.

Metabolic reprogramming is not only a consequence of immune stimulation but also plays an active role in determining immune cell activation. However, the metabolic pathways that contribute to vaccine responses have not been thoroughly characterized and represent an opportunity to increase vaccine responses in both healthy and immunosuppressed individuals. While the metabolic requirements of T cell and myeloid cells have been extensively studied (13–15), far less is known about how B cells support increased metabolic demands associated with differentiation, proliferation, and Ab production. This knowledge gap is particularly relevant in the context of immunosuppression, where metabolic pathways may be inadvertently disrupted in ways that compromise humoral immunity. To define the metabolic programs associated with effective B cell responses in successful vaccine responses in SOTRs, we utilized a combination of flow cytometry, single-cell RNA-seq (scRNA-seq), and functional analysis of metabolic activity. Using this approach, we identified a requirement for tight homeostatic regulation of lipid synthesis and fatty acid oxidation (FAO) in B cells, a balance that was unexpectedly altered by commonly used immunosuppressive drugs, including mycophenolate mofetil (MMF). Disruption of this lipid metabolic axis was associated with impaired B cell function and reduced vaccine responsiveness. Together, these findings reveal lipid metabolism as a critical and drug-sensitive regulator of human B cell immunity and suggest new avenues to enhance protective Ab responses without broadly relieving immunosuppression.

## Results

### SOTRs fail to respond to a 2-dose vaccine regimen.

Prior reports have shown that 2 doses of COVID-19 mRNA vaccines are sufficient to induce S-specific Abs in the majority of healthy controls (HCs), but not in SOTRs (7–11, 16). Consistent with previous reports, SOTRs enrolled in a national prospective, observational cohort had significantly lower anti–S IgG titers compared with HCs following the initial 2-dose regimen (Figure 1A). At peak response following 2 doses, 40% of SOTRs had titers below the positive Meso Scale Discovery (MSD) manufacturer’s seroconversion threshold, and the proportion of SOTRs who were seronegative increased to 80% during the waning period, 3–4 months following dose 2 (D2) (Figure 1A). Although HCs also demonstrated a significant decline in anti–S IgG titers approximately 6 months following their second dose, all HCs maintained IgG titers at or above the positive threshold at this time point (Figure 1A). The deficits in vaccine-induced immunity in SOTRs were most pronounced in class-switched S-specific Ab titers and B cells (Supplemental Figure 1, A–J; supplemental material available online with this article; https://doi.org/10.1172/JCI205170DS1). In contrast to class-switched B cells, there was no significant difference in the frequency of unswitched S-specific B cells (CD19^+^IgM^+^IgD^+^) or IgM titers between HCs and SOTRs (Supplemental Figure 1, B, H, and I). Notably, although only 20% of SOTRs had a positive anti-IgG response during the waning period following 2 vaccine doses, 76% had detectable class-switched S-specific B cells, indicating the possibility of boosting these B cells with a third vaccine dose (Supplemental Figure 1K). Therefore, we sought to determine mechanisms supporting or inhibiting S-specific B cell expansion in SOTRs who were able to respond to a third vaccine dose.

### Failure to respond to a third vaccine dose is associated with anti-metabolite treatment.

As has been previously demonstrated (7–10), a third vaccine dose significantly increased anti–S titers and S-specific B cell frequencies in SOTRs (Figure 1B), consistent with the CDC recommending 3 doses as a primary series for transplant recipients. Using a cohort of 44 SOTRs with matched before-and-after dose 3 samples (Supplemental Table 1), we identified a subset of SOTRs (32 of 44, 72.7%) considered responders on the basis of seroconversion after the third vaccine dose (Figure 1B and Supplemental Figure 2, A–G). There were no significant differences between responders and nonresponders in the vaccine received, organ transplanted, age, or time since transplantation (Supplemental Figure 2, H–L, and Supplemental Table 1). However, we found that all participants who failed to respond to a third dose received anti-metabolites as part of their immunosuppressive regimen (Figure 1C), specifically MMF or mycophenolic acid (MPA), which were commonly prescribed in combination with at least 1 other class of immunosuppressant (Figure 1D). MMF, a highly prescribed immunosuppressant used in approximately 93% of kidney transplant recipients and for autoimmune diseases (17, 18), is a prodrug of MPA, which inhibits inosine 5′-monophosphate dehydrogenase (IMPDH) to reduce de novo purine synthesis, limiting guanine availability and lymphocyte proliferation (19). SOTRs receiving MMF or MPA had significantly lower anti–S IgG titers relative to SOTRs who did not receive MMF or MPA. In addition, SOTRs receiving high-dose MMF or MPA (>1,000 mg/day or >720 mg/day, respectively) also showed a trend toward lower anti–S IgG titers relative to SOTRs receiving low-dose MMF or MPA (>1,000 mg/day or >720 mg/day, respectively) (Figure 1E). These findings are consistent with prior reports supporting a strong relationship between higher MMF dose and failure to respond to vaccination in several cohorts (20–23).

Notably, despite the association of MMF and MPA with a poor vaccine response, some participants receiving high- or low-dose MMF or MPA were responders after the third vaccine dose, with large heterogeneity in anti–S titers within each group (Figure 1E). We observed no significant differences between responders and nonresponders taking low or high-dose MMF in time since transplantation, age, or transplanted organ (Supplemental Figure 3), indicating that the response was not driven exclusively by these clinical parameters. Therefore, we aimed to uncover immunological pathways that could explain this heterogeneity of responses and improved immune cell function and vaccine responses in some individuals despite the presence of MMF or MPA. We specifically focused on understanding the immunometabolic pathways altered by this anti-metabolite and differences between vaccine responders and nonresponders who received these drugs.

### MMF directly influences immune cell lipid metabolism.

Mitochondrial bioenergetics were measured in PBMCs from SOTRs directly ex vivo via the Agilent Seahorse XF Cell Mito Stress Test in the absence of additional stimulation or exogenous substrates. The oxygen consumption rate (OCR) was used as a measurement of mitochondrial respiration/oxidative phosphorylation (OXPHOS). Unexpectedly, PBMCs from SOTRs receiving either low-dose MMF or other immunosuppression agents demonstrated substantially higher basal respiration compared with HCs (Figure 2A). In contrast, PBMCs from participants on high-dose MMF had a reduced OCR compared with PBMCs from the other SOTRs, indicating that this enhanced respiration was suppressed in SOTRs receiving high-dose MMF. A similar effect was seen in the extracellular acidification rate (ECAR), commonly used as a measurement of glycolytic activity, with SOTRs demonstrating increased ECAR compared with HCs (Supplemental Figure 4A).

To understand if this metabolic alteration was a direct effect of MMF or due to systemic activation following transplantation, we established an in vitro system in which PBMCs from healthy donors were treated with MPA. Although several studies have used patient plasma levels to propose in vitro doses of immunosuppressive drugs to replicate in vivo doses (estimated between 1 μM and 10 μM), pharmacokinetics studies of MPA have shown high variability and doses that most accurately represent the true levels of drug experienced by immune cells in tissue microenvironments are not known (24–27). We tested a range of doses and found that 5 μM MPA (referred to herein as high-dose) completely blocked proliferation of both T cells and B cells in vitro, whereas 0.05–0.15 μM MPA (low-dose) limited proliferation (Supplemental Figure 4B). Importantly, even high-dose MPA–treated cells maintained viability in culture (Supplemental Figure 4C), allowing us to study the effects of MPA at a range of doses in vitro. While patients are unlikely to receive doses that inhibit 100% of lymphocyte proliferation, we used these 2 doses to model extremes of MPA exposure, with doses received by patients likely falling between these extremes. Low-dose MPA increased both the OCR and ECAR compared with untreated PBMCs, reproducing the increased metabolic activity seen in PBMCs from SOTRs receiving low-dose MMF (Figure 2B and Supplemental Figure 4D). In contrast, PBMCs treated with high-dose MPA demonstrated reduced OCR compared with low-dose treatment, consistent with ex vivo data from SOTRs receiving high-dose MMF or MPA (Figure 2B). Although we focused primarily on MMF and MPA due to their association with poor vaccine responses, we found that tacrolimus, another commonly prescribed immunosuppressant, also increased OCR, whereas rapamycin did not (Supplemental Figure 4, E and F). These data indicate that certain immunosuppressive drugs can directly modulate immune cell metabolic activity, as reflected in our ex vivo analysis of PBMCs from SOTRs.

Although MMF and MPA have not been extensively investigated for their ability to modulate metabolic programs related to cellular energetics, a reported side effect of MMF and other commonly prescribed immunosuppressive medications is hyperlipidemia (28–30). Recent reports have also highlighted the importance of lipid metabolism in B cell function and generation of protective vaccine responses (31–33). Given these associations, we sought to understand whether MMF alters lipid metabolism in immune cells and what downstream effects MMF might have on vaccine responses. Low-dose etomoxir inhibits FAO by binding to CPT1a, the rate limiting enzyme of long chain FAO, and inhibiting transport of long-chain FAs into mitochondria without inducing off target effects on mitochondrial health (34–36). Low-dose MPA increased OCR primarily fueled by long chain FAO, as treatment with etomoxir completely reversed the increased OCR (Figure 2C). Conversely, we found that high-dose MPA–treated cells had minimal effects on the OCR with etomoxir treatment (Figure 2D) and were unable to oxidize long-chain FAs, as shown by measuring the OCR in response to supplemented palmitate (a long-chain FA) (Figure 2E). Cells treated with high-dose MPA had basal, ATP-linked, and maximal mitochondrial OCRs similar to those of untreated cells (Figure 2B) and maintained the same viability (Supplemental Figure 4C), indicating preserved mitochondrial integrity. However, high-dose MPA resulted in diminished mitochondrial oxidation of various supplemented substrates, including long- and medium-chain FAs, glucose, and glutamine (Figure 2F and Supplemental Figure 5, A–F), suggesting decreased substrate flexibility and impaired mitochondrial oxidative capacity. Importantly, PBMCs treated with MPA did not show overt signs of mitochondrial damage, as evidenced by their ability to respond to the mitochondrial stress test (Figure 2B), and there were no significant changes in membrane potential, as measured by tetramethylrhodamine ethyl ester (TMRE) or mitochondrial ROS, measured by MitoSOX (Supplemental Figure 5, G and H). Instead, consistent with a defect in FAO (37, 38), PBMCs from SOTRs on high-dose MMF or healthy PBMCs treated in vitro with high-dose MPA demonstrated the highest level of intracellular lipid droplet accumulation (Figure 2, G and H, and Supplemental Figure 5, I and J). Lipid droplets act as a mechanism to reduce lipid toxicity (39), but mitochondrial dysfunction due to excessive lipid accumulation in high-dose MPA–treated cells could explain the inability to increase the OCR in response to exogenous substrates. In contrast, the lower level of intracellular lipid accumulation driven by low-dose MPA (Figure 2, G and H) was probably the lipid source fueling FAO, as the Seahorse assays were performed in lipid-free media. Collectively, these data indicate that high-dose MMF resulted in the accumulation of intracellular lipid droplets and blunting of mitochondrial oxidation, whereas low-dose MMF resulted in more moderate lipid accumulation, with enhanced CPT1a-dependent mitochondrial lipid oxidation (Figure 2I).

### Low-dose MPA induces CPT1a-dependent FAO in B cells but not T cells.

We next sought to understand the relative contribution of the 2 main cellular targets of MPA, B and T cells, to the increased FAO observed in total PBMCs. Because of the low cell counts in primary SOTR PBMCs, we utilized our in vitro system with high- and low-dose MPA treatment. Consistent with increased CPT1a expression in B cells compared with T cells (Figure 3A), only B cells increased their OCR in response to low-dose MPA (Figure 3B). Similar to total PBMCs, high-dose MPA blunted the increase in the OCR compared with low-dose MPA in B cells (Figure 3, B and C). Inhibition of CPT1a by etomoxir significantly reduced the OCR to baseline levels in B cells but had no effect on T cells (Figure 3, D and E). Even in the presence of polyclonal B cell stimulation with cytosine-phosphorotioate-guanosine (CpG), which should increase cellular demands for lipids, MPA led to lipid accumulation in B cells (Figure 3F) as well as T cells and monocytes (Supplemental Figure 6, A and B). In the presence of polyclonal CpG stimulation, total lymphocytes and B cells showed increased mitochondrial ROS only in the presence of high-dose MPA, demonstrating that continued exposure to high-dose MPA induced mitochondrial stress in B cells (Figure 3G).

To determine whether these changes in lipid metabolism and mitochondrial stress were an on-target effect of MPA or were reflective of proliferation blockade and/or cellular stress, B cells were stimulated in the presence of MPA and supplemented with guanosine to restore GMP and GTP levels. Exogenous guanosine reduced proliferation in untreated and low-dose MPA–treated B cells but rescued proliferation in high-dose treated B cells, as expected (Figure 3H). Interestingly, guanosine supplementation reduced total B cell lipid accumulation in all treatment groups (Figure 3I) and in T cells and monocytes that were not stimulated to proliferate (Supplemental Figure 6, C and D). To further dissect the role of proliferation versus inhibition of de novo purine synthesis, we stimulated B cells in the presence of 6-MP (the active component of azathioprine), which inhibits de novo purine synthesis through alternative mechanisms, limiting both GTP and ATP production (Supplemental Figure 6, E–H). Although 6-MP was less effective at inhibiting proliferation at doses equivalent to those of MPA, it increased lipid accumulation in B cells, T cells, and monocytes that was reversed with the addition of guanosine (Supplemental Figure 6, F–H). These data suggest that cellular levels of GMP and GTP are directly linked to lipid accumulation in cells and that lipid accumulation is not simply a reflection of inhibited proliferation.

To corroborate our findings that SOTRs receiving MMF had altered B cell lipid metabolism, we isolated B cells from SOTRs and HCs for ex vivo lipidomics analysis. Principle component analysis (PCA) of lipid profiles revealed segregation by lipid species, with SOTRs receiving low- or high-dose MMF showing increased triglyceride (TAG) accumulation (Figure 4, A and B). SOTR2 receiving high-dose MMF showed the highest levels of the diacylglycerol (DAG) DG 16:0_26:0 and several TAG species. SOTR4, who was not taking MMF, had the lowest levels of TAG accumulation among SOTRs. DAG serves as a precursor for TAG, which is commonly stored in lipid droplets, or for phospholipids, which are major components of cell membranes (Figure 4C) (40). Compared with HCs, SOTRs demonstrated high levels of TAG accumulation and lower levels of several phospholipids, such as phosphatidylserines (PS) and phosphatidylethanolamines (PE). Overall, B cells derived from SOTRs showed an increase in DAG production, enhanced TAG synthesis and storage, and decreased phospholipid synthesis (Figure 4C). Combined with the results from our in vitro model (Figure 3, F and I), these data indicate that both low- and high-dose MMF directly promoted intracellular TAG accumulation through depletion of intracellular guanosine nucleotides. Importantly, low-dose MMF–treated B cells could utilize these TAGs for FAO in a CPT1a-dependent manner, whereas high-dose MMF–treated B cells could not.

### CPT1a^+^CD11c^+^ B cells are associated with vaccine responses.

To understand how immunosuppression-induced alterations in B cell lipid metabolism affect specific B cell subsets and the response to vaccination, we designed a high-parameter flow cytometry panel to interrogate metabolic programs in B cells at single-cell resolution before and after vaccination. Using dimensionality reduction via uniform manifold approximation and projection (UMAP), with unbiased clustering (41), we identified 6 unique clusters of B cells (Figure 5, A and B). Cluster 4 contained S-specific B cells and was increased in responders after dose 3 (Figure 5, B and C). Cluster 3 was significantly increased in responders before and after dose 3 and was defined by coexpression of CD11c and CPT1a (Figure 5, B and C). CPT1a is the rate-limiting enzyme in long-chain FAO, suggesting that these B cells were primed to support FAO. CD11c expression has been associated with atypical memory and extrafollicular B cells (42, 43), which are traditionally linked to chronic antigenic stimulation or autoimmunity (42), but more recent studies show that CD11c^+^ B cells are more diverse and can contribute to vaccine responses in healthy individuals (44–48). Together, these data demonstrate that a subset of SOTRs responded to a third vaccine dose with expansion of B cells expressing CD11c and CPT1a.

Given the distinct nature of cluster 3, we devised a manual gating scheme to evaluate this cell population in relation to other canonical B cell subsets (Supplemental Figure 7, A and B). We found that CD11c^+^ B cells derived from both HCs and SOTRs uniformly expressed higher levels of CPT1a, indicating that this expression pattern was an inherent property of CD11c^+^ B cells (Figure 5D). Consequently, gating on total CD11c^+^ B cells identified a cell population that was highly reminiscent of cluster 3. The frequency of CD11c^+^ B cells significantly correlated with the frequency of cluster 3 B cells (Figure 5E) and largely recapitulated the unbiased clustering (Figure 5F and Supplemental Figure 7, A and B). Both CD11c^+^ B cells and cluster 3 B cells were predominantly double-negative (DN) (IgD^–^CD27^–^) B cells and activated naive (IgD^+^, CD11c^+^, CD86^+^) B cells (49, 50). However, CD11c^+^ B cells were also found within the classical memory (CD27^+^) and CD27^–^ memory (CD24^+^CD38^–^) B cell compartments (Figure 3F). Rather than being defined by a specific cellular compartment, these cells were unified by their expression of CD11c and CPT1a and existed in multiple B cell activation and differentiation states. Thus, gating on total CD11c^+^ B cells allowed us to evaluate CPT1a^+^ B cells in a larger number of participants than had been included in the unbiased screening to understand their relationship to the vaccine response.

We next evaluated the frequency of CD11c^+^ B cells in 2 distinct cohorts of SOTRs (Supplemental Figure 8, A–D). The initial cohort (Figure 1) was an observational cohort in which SOTRs were assessed before and after vaccination irrespective of outcome of the 2-dose vaccine series (Supplemental Figure 8, A and B), and the second cohort studied was a single-arm, open-label clinical trial assessing the safety and immunogenicity of a third mRNA vaccine dose in kidney transplant recipients with negative or low-titer anti-receptor–binding domain Abs after 2 vaccine doses (NCT04969263, Supplemental Table 2 and Supplemental Figure 8, C and D). CD11c^+^ B cell frequencies were comparable between both cohorts allowing us to also evaluate the cohorts together (Figure 5, G and H). Consistent with the frequencies of cells identified as cluster 3, CD11c^+^ B cells were expanded in SOTR responders and less frequent in HCs (Figure 5G). Across the 2 cohorts, we also found a negative correlation between the MMF or MPA dose and the percentage of CD11c^+^ B cells in kidney recipients, suggesting that high-doses of MMF/MPA restrict the expansion of CD11c^+^CPT1a^+^ B cells (Supplemental Figure 8, E and F). The lack of significant correlation in nonkidney recipients may reflect differences in baseline immunology and concomitant immunosuppressive regimens across the 2 groups. We then evaluated the contribution of CD11c^+^ B cells to the S-specific pool before and after vaccination. While the frequencies of S-specific B cells that expressed CD11c were low before vaccination, the frequency increased to approximately 50% of the total S-specific pool following vaccination in both HCs and SOTR responders (Figure 5H). SOTR nonresponders had frequencies of S-specific B cells that were too low to evaluate phenotype accurately. These data are consistent with recent reports highlighting the prevalence of CD11c^+^ B cells within S-specific B cells following both SARS-CoV-2 infection and vaccination of HCs (45–47).

We next compared anti–S IgG Ab titers and CD11c expression on total and S-specific B cells at peak and waning time points after doses 2 and 3 (Figure 5, I and J). HCs maintained high Ab titers after 2 doses that were further boosted by dose 3, with peak titers associated with a transient increase in CD11c expression on S-specific B cells after D2 (Figure 5I). In contrast, SOTRs generated few S-specific B cells after 2 doses (Supplemental Figure 1, E and F) and failed to expand CD11c^+^ S-specific B cells, corresponding with lower and heterogeneous peak Ab titers (Figure 5J). After dose 3, SOTRs showed increased CD11c expression within the S-specific B cell pool, which was associated with higher and more sustained Ab titers (Figure 5J). Consistent with a role in class-switched vaccine responses, S-specific CD11c^+^ B cells had higher IgG expression and somatic hypermutation than did CD11c^−^ S-specific B cells (Supplemental Figure 8, G and H), although both CD11c^+^ and CD11c^−^ S-specific B cell frequencies correlated with anti–S IgG titers in SOTRs (Supplemental Figure 8I). After magnetic isolation, both CD11c^+^ and CD11c^−^ B cells produced anti–S IgG following polyclonal stimulation in vitro (Supplemental Figure 8, J and K). Together, these data contribute to the growing body of evidence that CD11c^+^ B cells play a previously underappreciated role as a component of the vaccine response. CD11c^+^ B cells appear to have a role in early vaccine-induced Ab production in both HCs and SOTRs, while our data suggest that the association between baseline CD11c^+^ B cell frequency and vaccine response was unique to SOTRs.

### CD11c^+^ B cells are metabolically active and maintain the ability to differentiate into plasmablasts in the presence of MPA.

To understand relevant pathways that could support vaccine responses in CD11c^+^ B cells, we performed scRNA-seq to analyze transcriptional profiles of CD11c^+^ B cells in SOTRs and HCs before and after vaccination (Supplemental Table 3). By comparing gene set enrichment analysis (GSEA) of CD11c^+^ and CD11c^–^ B cells, we found that several of the top gene sets that were significantly enriched in CD11c^+^ B cells were associated with metabolic activity (Figure 6A). Of note, CD11c^+^ B cells had higher expression of gene sets associated with OXPHOS, mTOR signaling, FA metabolism, and glycolysis (Figure 6B). These profiles were consistent with our flow cytometry demonstrating high expression of CPT1a protein in CD11c^+^ B cells (Figure 6C). Given our earlier discovery that low-dose MPA induced CPT1a-dependent FAO in B cells, these metabolic profiles suggest that CD11c^+^ B cells should preferentially increase FAO compared with CPT1a^lo^/CD11c^–^ counterparts. Because the frequency of CD11c^+^ B cells was too low to accurately measure the OCR by Seahorse assay, we took advantage of a fluorescent FA analog (FAO-blue) that optimally emits fluorescence after undergoing β-oxidation within the mitochondria (Figure 6, D and E). As expected, T cells did not show an increase in FAO-blue signal in response to MPA, whereas B cells did. When segregating the B cells according to CD11c expression, we found that CD11c^+^ B cells had increased FAO-blue signal ex vivo. In agreement with mitochondrial stress testing of isolated B cells, we found that CD11c^+^ B cells had increased FAO-blue signal after in vitro culturing with low-dose MPA but not high-dose MPA (Figure 6E). Interestingly, CD11c^–^ B cells did not experience increased FAO in response to MPA treatment (Figure 6E). Of note, monocytes also expressed CPT1a (Supplemental Figure 9, A–C) and showed trends of increasing lipid oxidation in response to MPA (Supplemental Figure 9D), potentially contributing to the increased OCR seen in the bulk PBMC Seahorse assays. Together, these data suggest that CD11c^+^ B cells were inherently capable of oxidizing lipids, with greatest enhancement of FAO-blue signal upon treatment with low-dose MPA.

The ability of a cell to rapidly proliferate and differentiate is reliant on its ability to support increased metabolic demands through enhanced ATP production. Therefore, we hypothesized that CD11c^+^ B cells would be better equipped to support plasmablast differentiation by utilizing enhanced FAO in the presence of MPA. This would provide a salvage mechanism to enable B cell differentiation even in the presence of immunosuppression. To test this hypothesis, CD11c^+^ or CD11c^–^ B cells from HCs were sorted and subjected to polyclonal stimulation in the presence of low- or high-dose MPA (Figure 6, F and G). Consistent with previous reports (48, 51–53) and the metabolically active transcriptional profiles, we found that CD11c^+^ B cells were more able to differentiate into plasmablasts in the absence of MPA (Figure 6G). Remarkably, CD11c^+^ B cells demonstrated significantly higher levels of plasmablast differentiation in the presence of low-dose MPA compared with CD11c^–^ B cells. CD11c^–^ B cell differentiation into plasmablasts was almost completely abrogated in the presence of even low-dose MPA, which had been titrated to allow for limited proliferation of total B cells. However, neither CD11c^+^ nor CD11c^–^ B cells were able to undergo plasmablast differentiation in the presence of high-dose MPA. These data suggest that CD11c^+^CPT1a^+^ B cells can metabolize FAs and differentiate into plasmablasts in the presence of low- but not high-dose MPA.

### Reducing lipid synthesis promotes plasmablast differentiation in the presence of MPA.

While CD11c^+^CPT1a^+^ B cells provide a mechanism to respond to vaccination, our data also show that high-dose MPA induced lipid accumulation and reduced mitochondrial function. These findings suggest that variation in lipid metabolism may contribute to heterogeneity in vaccine responses despite similar MPA or MMF doses (Figure 1E) and may offer a targetable pathway to improve B cell responses without reducing immunosuppression. However, the in vivo mechanisms driving lipid accumulation in B cells remain unclear and may involve either increased uptake or synthesis. We therefore selected a limited set of lipid metabolism gene sets (Supplemental Table 4) to examine transcriptional differences in total B cells using the scRNA-seq dataset. We performed GSEA at the single-cell level, comparing B cells from SOTRs versus HCs, SOTRs on high- versus low-dose MMF or MPA, and SOTR responders versus nonresponders to vaccination (Figure 7A). We saw the largest number of significantly enriched gene sets comparing HC to SOTRs (Figure 7A), with the greatest differences in gene sets related to cholesterol biosynthesis and homeostasis (Figure 7B). The Hallmark Cholesterol Biosynthesis gene set includes several genes associated with both cholesterol and FA synthesis, suggesting the accumulation of lipids was driven by de novo synthesis within B cells. Confirming these findings, we found that MPA treatment did not enhance the uptake of the fluorescently labeled long-chain FA BODIPY-C16 in unstimulated or CpG-stimulated conditions (Supplemental Figure 10, A and B). Instead, increased lipid synthesis and lipolysis may be fueled by enhanced glucose uptake (Supplemental Figure 10C) and glucose metabolism (Supplemental Figure 4, A and D), as has been demonstrated previously for memory CD8^+^ T cells (54). We also found that low-dose MPA increased glucose-6-phosphate dehydrogenase (G6PDH) activity, suggesting increased flux from glucose into the pentose-phosphate pathway (PPP) (Supplemental Figure 10D). Furthermore, inhibition of G6PDH significantly decreased lipid accumulation in CPG-stimulated low-dose MPA–treated B cells (Supplemental Figure 10E). Consistent with our in vitro data, we found that SOTRs taking low-dose MMF or MPA had increased gene signatures associated with mitochondrial FA β-oxidation compared with those taking high-dose MMF or MPA (Figure 7C). However, high-dose MMF or MPA was associated with even greater enrichment of the cholesterol homeostasis gene set, consistent with increased lipid accumulation and further supporting the notion of lipid synthesis and accumulation occurring over a dose-dependent continuum. Finally, nonresponders to vaccination showed increased enrichment in gene sets associated with lipid particle organization and transport (Figure 7D), potentially reflecting increased toxicity due to lipid accumulation.

Individual genes from the lipid gene sets were next probed for significant differences across the same comparator groups to identify therapeutic targets (Figure 7, E–G). We identified several genes associated with cholesterol synthesis that were increased in SOTRs compared with HCs, including *SREBF2*, which encodes SREBP2, a master regulator of lipid synthesis. While SREBP2 can lead to increased FA synthesis, it preferentially induces cholesterol synthesis (55, 56). The rate-limiting enzyme of polyamine synthesis, *ODC1*, was also significantly enriched in nonresponders to vaccination compared with responders (Figure 7G). Although *ODC1* has been extensively characterized for its role in polyamine synthesis, the exact relationship to lipid metabolism is not as well defined. However, it has been reported to increase cholesterol synthesis through hypusination and activation of eIF5A, which supports the translation of *SREBF2* (57). Although cholesterol is required for a variety of cellular functions during differentiation, it is an energetic dead end in B cells and cannot be catabolized for ATP production (58). Excess cholesterol accumulation in B cells can inhibit mitochondrial function and lead to a redox imbalance (59), potentially contributing to enhanced lipid toxicity and B cell nonresponse.

We identified inhibitors of SREBP (fatostatin), FA synthase (C75), cholesterol synthesis (atorvastatin), and cholesterol/polyamine synthesis (difluoromethylornithine [DFMO]) to determine whether blocking different aspects of lipid synthesis could prevent excess lipid accumulation and toxicity (Figure 8A). PBMCs were stimulated with CpG for 6 days in the presence of low- or high-dose MPA and treated with the above-described inhibitors (Figure 8, B and C). All inhibitors were titrated to have minimal effects on untreated cells to prevent global inhibition of lipid synthesis and B cell proliferation. All inhibitors tested in vitro at optimized doses demonstrated trends toward increased plasmablast differentiation in the presence of low-dose MPA. Atorvastatin and DFMO showed a significant enhancement of plasmablast differentiation in both low- and high-dose MPA treatment, although the effect was more modest with high-dose MPA due to the profound proliferation inhibition (Figure 8). Although atorvastatin and DFMO were able to enhance plasmablast differentiation, they did not significantly increase proliferation of MPA-treated cells (Figure 8D). Atorvastatin led to a significant reduction in lipid accumulation in B cells treated with low-dose MPA, to levels similar to those in guanosine-treated cells, but failed to significantly lower lipids in high-dose MPA–treated cells (Figure 8E and Supplemental Figure 10F). Although not addressed in the present study, MPA-driven cholesterol synthesis and accumulation may also inhibit the function of other immune cells that support B cell function, and we found that atorvastatin was similarly able to reduce lipid accumulation in T cells and monocytes (Supplemental Figure 10, G and H). Importantly, we found that isolated B cells still showed increased plasmablast differentiation when low-dose MPA–treated cells were supplemented with atorvastatin, although to a lesser degree than when cultured in the presence of other immune cells (Figure 8, F and G). Consistent with previous reports showing a decrease in B cell activation in the presence of statins (60), higher doses of atorvastatin inhibited plasmablast differentiation (Figure 6G). Given the high prevalence of dyslipidemia and statin use among SOTRs (28–30), we investigated whether statin use improved vaccine responses. We observed a trend toward increasing anti–S IgG titers, plasmablast frequencies, and vaccine responses in SOTRs prescribed statins while on MMF or MPA compared with SOTRs not prescribed statins (Figure 8, H–J). Together, these data suggest that targeting cholesterol synthesis through FDA-approved drugs such as statins or DFMO may lead to improved B cell function and vaccine responses in SOTRs experiencing MMF- or MPA-induced lipid toxicity across multiple doses.

### Measurements of lipid metabolism outperform standard clinical parameters in predicting vaccine responses.

Although we observed trends toward increased anti–S IgG titers and plasmablasts in SOTRs receiving MMF or MPA and prescribed statins (Figure 8, H and I), these were univariate analyses that did not account for multiple other clinical factors that could influence the vaccine response. Therefore, to more comprehensively evaluate the contribution of lipid metabolism to the heterogenity of vaccine responses in the presence of MPA, we selected individuals taking MMF or MPA for whom data on statin usage was available to measure clinical lipid panels (*n* = 65; 44 responders, 21 nonresponders). These measurements were combined with basic clinical features (organ transplanted, time since transplantation, age, sex, immunosuppression regimen) and used in a random forest model to identify features most associated with vaccine responses (Figure 9A). Receiver operating characteristic (ROC) analysis demonstrated high discriminative ability of the random forest model (AUC: 0.853, 95% CI: 0.762–0.944; Figure 9B), and the performance metrics summarized in Figure 9C further showed 95.4% accuracy, 85.7% sensitivity, 100% specificity, and an F1 score of 0.92. The top contributing variable in the model was receiving a lung transplant, as all 3 lung transplant recipients were nonresponders (Figure 9D), followed by the total daily dose of MMF or MPA (Figure 9E). Interestingly, the next 4 variables (triglycerides, cholesterol/HDL ratio, cholesterol, and statin usage)were all related to lipid metabolism (Figure 9, F–I), outperforming several clinical factors known to associate with vaccine responses, such as time since transplantation, age, and use of other immunosuppressive drugs (61, 62). Higher triglyceride and cholesterol levels as well as higher and cholesterol/HDL ratios were all associated with a nonresponse, whereas statin use was associated with a response. To directly address whether clinical heterogeneity (organ type, age, sex, time since transplantation, immunosuppressive regimen, comorbidities) confounded our findings, we performed a sensitivity analysis comparing 6 model variants (Supplemental Figure 11). Critically, lipid parameters plus MMF dose alone performed nearly identically to the full model (AUC: 0.865 vs. 0.853), whereas clinical variables alone showed poor discrimination (AUC: 0.503), and the MMF dose alone was insufficient (AUC: 0.557). This demonstrates that pretreatment lipid metabolism, rather than clinical heterogeneity or drug dosing, drives vaccine response prediction. Together, these data demonstrate an association between increased systemic lipids and vaccine nonresponse. However, given the relatively small sample size and the limited number of lung transplant recipients, the machine-learning findings should be interpreted as exploratory, and independent validation will be required to confirm the robustness of the performance metrics and feature rankings. Although correlative in nature, these data are supported by in vitro findings and warrant future controlled studies to evaluate blockade of cholesterol synthesis as a method to improve the vaccine response in SOTRs taking MMF or MPA.

## Discussion

To improve outcomes in immunosuppressed individuals with poor vaccine responses, it is critical to understand how protective humoral immunity is preserved despite immunosuppression. Using COVID-19 vaccination as a model in SOTRs receiving diverse immunosuppressive regimens, we identified B cell lipid metabolism as a targetable regulator of early vaccine response. Immunosuppressive therapy, particularly with MMF or MPA, increased B cell lipid synthesis and accumulation through guanosine nucleotide depletion. Effective B cell differentiation and Ab production required balancing lipid synthesis with FAO, which was achieved either through recruitment of CPT1a^+^CD11c^+^ B cells or pharmacologic inhibition of cholesterol synthesis.

MMF and MPA have been widely associated with poor vaccine responses (20–23), although as demonstrated by our data, individuals receiving the same dose of MMF may have had highly variable vaccine responses that cannot be explained by standard clinical features alone. To explore potential mechanisms underlying MPA’s association with a poor vaccine response, we developed a controlled in vitro model in which healthy immune cells were treated with high or low doses of MPA. Clinically, immunosuppressive medication doses are chosen on the basis of graft function, autoimmune disease activity, or blood drug levels, but these approaches are neither sensitive nor specific for assessing the degree of immunosuppression, particularly for MMF, for which blood levels are not routinely measured (63). By integrating ex vivo samples and a range of in vitro doses, we captured metabolic programs that may more accurately reflect the level of immunosuppression of SOTR immune cells in vivo. Using this model, we found increased metabolic activity and OCRs in immune cells following low-dose MPA treatment, while high-dose MPA suppressed oxidative metabolism, consistent with prior reports using higher concentrations (64). Strikingly, both doses promoted lipid synthesis and accumulation, suggesting a continuum in which lipid synthesis may initially support increased FAO, but excessive accumulation drives mitochondrial stress. This aligns with limited reports of MPA-induced lipid droplet formation in epithelial, breast cancer, and T cell or monocytic lines (65–68). These findings are also consistent with increased evidence of metabolic disorders including dyslipidemia in SOTRs (28–30). Although our data may suggest that some of these metabolic syndromes are driven at least in part from off the off-target effects on lipid metabolism of these drugs, a limitation of our study is that we did not gather detailed clinical data on comorbidities and therefore cannot directly address this hypothesis. Further work is also needed to fully define the reliance of B cells on CPT1a-mediated FAO in the presence of MPA through genetic knockout and isotope-tracing–based approaches.

Mechanistically, we found that guanosine supplementation reversed lipid accumulation, directly linking nucleotide depletion to lipid metabolism. While the exact mechanism driving lipid synthesis is not fully defined, one possibility is that IMPDH inhibition increases flux through the PPP to restore guanosine, generating excess NADPH that fuels lipid synthesis (69). Increased flux may also elevate AMP, activating AMPK and enhancing FAO (70). While further work is required to fully delineate this mechanism, our data reveal that MMF and MPA exert dose-dependent effects on immune cell metabolism through altered nucleotide and lipid balance. The ways in which this dysregulated lipid metabolism alters B cell function and vaccine response had not, to our knowledge, been previously explored.

B cells require substantial energy to sustain proliferation, class-switching, and Ab production, but the specific pathways supporting these increased metabolic demands have been ill defined (71, 72). Mouse studies have implicated both FAO and glycolysis in the support of B cell function (73–76). Despite conflicting results in mice, lipid homeostasis and cholesterol metabolism have recently been identified as metabolic regulators of vaccine responses in humans and mice (31, 32). In an early example of metabolic profiling after vaccination, Li et al. integrated plasma metabolomics, PBMC transcriptomics, cell phenotypes, and cytokines after Zostavax in healthy adults, identifying sterol/lipid pathways (particularly SREBP1 and its targets), along with inositol phosphate and glycerophospholipid metabolism as key integrators of Ab and T follicular helper cell (Tfh) responses (31). Follow-up studies established that sterol biosynthesis is required for effective vaccine responses, as SREBP inhibition in mice and statin treatment in healthy humans both reduced Ab titers following vaccination (32, 33, 77–79). Our work extends these findings, but describes a paradigm in humans in which excessive lipid biosynthesis can inhibit B cell function and vaccine responses. Our data suggest that a fine balance of lipid metabolism is required for fully functional B cells, with lipid synthesis functioning as a metabolic rheostat, whereby both excessive and insufficient lipid synthesis impairs B cell responses, but an intermediate level supports optimal function. Thus, while concordant with Li et al. in highlighting lipid networks as central to vaccine responses, our data refine the model by demonstrating that immunosuppressant-driven metabolic stress can pathologically shift these pathways to blunt B cell immunity in SOTRs. Although identified here in the context of MMF and organ transplantation, this is likely a conserved pathway critical for B cell biology and may also influence B cell immunity in other conditions associated with hyperlipidemia, such as obesity, HIV, and autoimmunity.

We identified a critical role for CPT1a^+^CD11c^+^ B cells in supporting vaccine responses in the presence of increased lipid synthesis by promoting lipid homeostasis through increased FAO. Although CD11c^+^ B cells, often referred to as atypical, inflammatory, extrafollicular, double negative (DN), or age-associated B cells (ABCs), have been described in autoimmunity, chronic infection, and repeated antigen exposure (42, 80–84), their role in protective humoral immunity remains less clear. Our data expanded upon a previous study that characterized CD11c^+^ B cells as having elevated expression of metabolic enzymes associated with OXPHOS and FAO and demonstrated increased metabolic activity and FAO capacity at the transcriptional, protein, and functional levels (85). The enhanced metabolic activity enabled CD11c^+^ B cells to maintain their differentiation capacity, even in the presence of low-dose MPA, whereas CD11c^–^ B cells were more susceptible to the inhibitory effects of MPA. CD11c^+^ B cells were more abundant in SOTRs who responded to vaccination, suggesting that a more favorable lipid metabolic state may permit their accumulation. The transient expansion of CD11c^+^ B cells within the S-specific response is consistent with prior reports following COVID-19 vaccination (47) and suggests that their metabolic properties may be important for rapid early responses rather than sustained memory. Because participants in our cohort were boosted frequently during the pandemic, we were not able to fully assess long-term memory responses. These findings highlight the importance of defining the metabolic requirements of early versus sustained B cell responses for next-generation vaccine design. Notably, our ex vivo data were generated from samples collected early in the pandemic, before hybrid immunity became widespread, and all participants had vaccine-induced immunity only. This allowed us to focus on de novo responses to a novel antigen; however, in the current setting of repeated boosting and preexisting immunity, memory B cell pools may play a greater role in shaping the response, potentially altering the relative contribution of CD11c^+^ B cells.

In SOTRs with low frequencies of lipid-oxidizing CD11c^+^CPT1a^+^ B cells, strategies to augment vaccine responses while maintaining transplant immunosuppression are needed. Our data suggest that limiting cholesterol synthesis with well-tolerated drugs such as statins may promote B cell lipid homeostasis under immunosuppression. Although prior studies reported reduced vaccine responses in healthy statin users (77–79), this contrast may reflect distinct metabolic requirements in SOTRs. Because statins are widely prescribed in this population, controlled trials are needed to test whether cholesterol synthesis inhibition enhances vaccine responses, particularly across MMF and MPA dose ranges.

In summary, our findings reveal the importance of lipid metabolism in driving B cell–mediated vaccine responses and the influence of immunosuppressive regimens on this homeostatic balance. These findings can be used to design vaccine formulations for both immunocompetent and immunosuppressed individuals.

## Methods

### Sex as a biological variable.

Both male and female participants were included in this study. Sex was considered as a biological variable in the experimental design and analysis. As shown in Supplemental Tables 1–3, there were no significant differences between male and female participants in responder/nonresponder classifications.

### Study design and participant recruitment.

SOTRs were enrolled in a national prospective, observational cohort, as previously described (7, 8, 20). All SOTR participants were recruited virtually, provided detailed transplant history, and gave oral informed consent under an IRB-approved waiver of written consent. Vaccines were administered independently in the community. To evaluate alternative immunization strategies, the COVID-19 Protection After Transplant (CPAT) pilot trial (ClinicalTrials.gov NCT04969263) was launched as a single-arm, open-label trial testing Ab and cellular responses to an additional (third) primary dose of homologous mRNA vaccination in kidney transplant recipients with a history of negative (<0.8 units/mL) or low (0.8–50 units/mL) titers on the Roche Elecsys anti–SARS-CoV-2 S assay. HC participants were enrolled under institutional approvals from Johns Hopkins University and Emory University following written informed consent. All patient samples were deidentified prior to processing and testing. Participants with a prior clinical history of SARS-CoV-2 or detectable anti-nucleocapsid Abs indicative of prior natural infection were excluded from the analysis.

### Demographic information.

Demographic identities, including biological sex, race, and ethnicity, were self-reported by participants, who chose from fixed categories defined by the investigators in alignment with NIH guidelines. See Supplemental Tables 1–3 for a breakdown of the demographics for each cohort. For deidentified healthy donor leukopaks, donor-level clinical data beyond general health screening and biological sex were unavailable.

### Statistics.

Statistical analyses were performed using Prism 10 (GraphPad Software) and R (version 4.3.2). Data are presented as mean ± SEM unless otherwise noted. Comparisons between paired observations were performed using the Wilcoxon matched-pairs signed-rank test, where appropriate. Comparisons between independent groups were performed using the Mann-Whitney *U* test or Kruskal-Wallis test, as indicated in the figure legends. Comparisons involving two experimental factors were performed using 2-way analysis of variance (ANOVA). Comparisons of enrichment scores between groups were performed using Welch’s *t* test, with *P* values adjusted using the Benjamini-Hochberg method where indicated. Correlations were assessed using Spearman’s rank correlation. Differential gene expression analysis was performed using a 2-tailed Wilcoxon rank-sum test implemented in Seurat, with *P* values adjusted using Bonferroni correction. For high-dimensional classification, a random forest model was trained using the caret package in R with repeated 20-fold cross-validation. Discriminative performance was evaluated using receiver operating characteristic (ROC) analysis and the area under the curve (AUC), calculated using the pROC package. A *P* value of less than 0.05 was considered statistically significant. Detailed experimental workflows, assay conditions, Ab panels, and custom computational workflows are provided in the Supplemental Methods.

### Study approval.

Human clinical studies were approved by the Johns Hopkins University IRB (Baltimore, Maryland, USA; approvals IRB00248540 and IRB00027183) and the Emory University IRB (Atlanta, Georgia, USA; approval IRB00002061). All participants or their legal guardians provided oral or written informed consent prior to study inclusion, in accordance with the Declaration of Helsinki.

### Data availability.

All data needed to evaluate the conclusions in this study are present in the manuscript, the supplemental materials, and/or the uploaded Supporting Data Values file. The scRNA-seq data are available in the NCBI Gene Expression Omnibus (GEO) database (GEO GSE341347). All data are available upon request.

## Manuscript information

### Author contributions.

EAT, AHK, DLS, WAW, ALC, and JRB conceptualized the study and contributed to initial cohort development. EAT, AF, KR, K Redinger, LS, SBW, NES, SD, SL, LB, and HK contributed to methodology development and data generation. EAT prepared the figures and performed visualizations. SLK, DLS, WAW, ALC, and JRB acquired funding for the study. YE and AART assisted with project administration. EAT, SLK, DLS, AART, WAW, ALC, and JRB supervised the study. LAS, RDL, PSH, MAR, CPL, ELP, EJP, HK, SLK, NR, JNB, DLS, AART, WAW, AHK, and JRB contributed to critical discussion and interpretation of the data. EAT wrote the original draft of the manuscript, with input from ALC and JRB. EAT, AF, K Roznik, NES, SD, SL, LB, LAS, LS, K Redinger, SBW, SLK, NR, JNB, YE, RDL, PSH, MAR, CPL, ELP, EJP, HK, AHK, DLS, AART, WAW, ALC, and JRB reviewed and edited the manuscript. All authors reviewed and approved the final manuscript.

### AI disclosure.

The authors declare that no AI tools were used in the original research for or writing of this article.

### Conflict of interest.

ET, AC, and JB are co-inventors on a provisional patent (no. 18/855,981) related to work described in this manuscript.

### Funding.

This study was supported by the following:

- Ben-Dov Family (to DLS).
- National Cancer Institute (NCI), NIH grant U54CA260492 (to ALC and SLK).
- National Institute of Allergy and Infectious Diseases (NIAID), NIH grant K24AI144954 (to DLS).
- NIAID, NIH grant K08AI156021 (to AHK).
- NIAID, NIH grant K23AI157893 (to WAW).
- NIAID, NIH grant U01AI138897 (to DLS).
- National Institute of Diabetes and Digestive and Kidney Diseases (NIDDK), NIH grant K01DK101677 (to ABM).
- Johns Hopkins Vice Dean of Research Support for COVID 19 research in Immunopathogenesis (to ALC).
- NIAID, NIH grant K22AI175398 (to EAT).

This work is the result of NIH funding, in whole or in part, and is subject to the NIH Public Access Policy. Through acceptance of this federal funding, the NIH has been given a right to make the work publicly available in PubMed Central.

## Supplementary Material

Supplemental data

Supporting data values

## Figures and Tables

**Figure 1:**
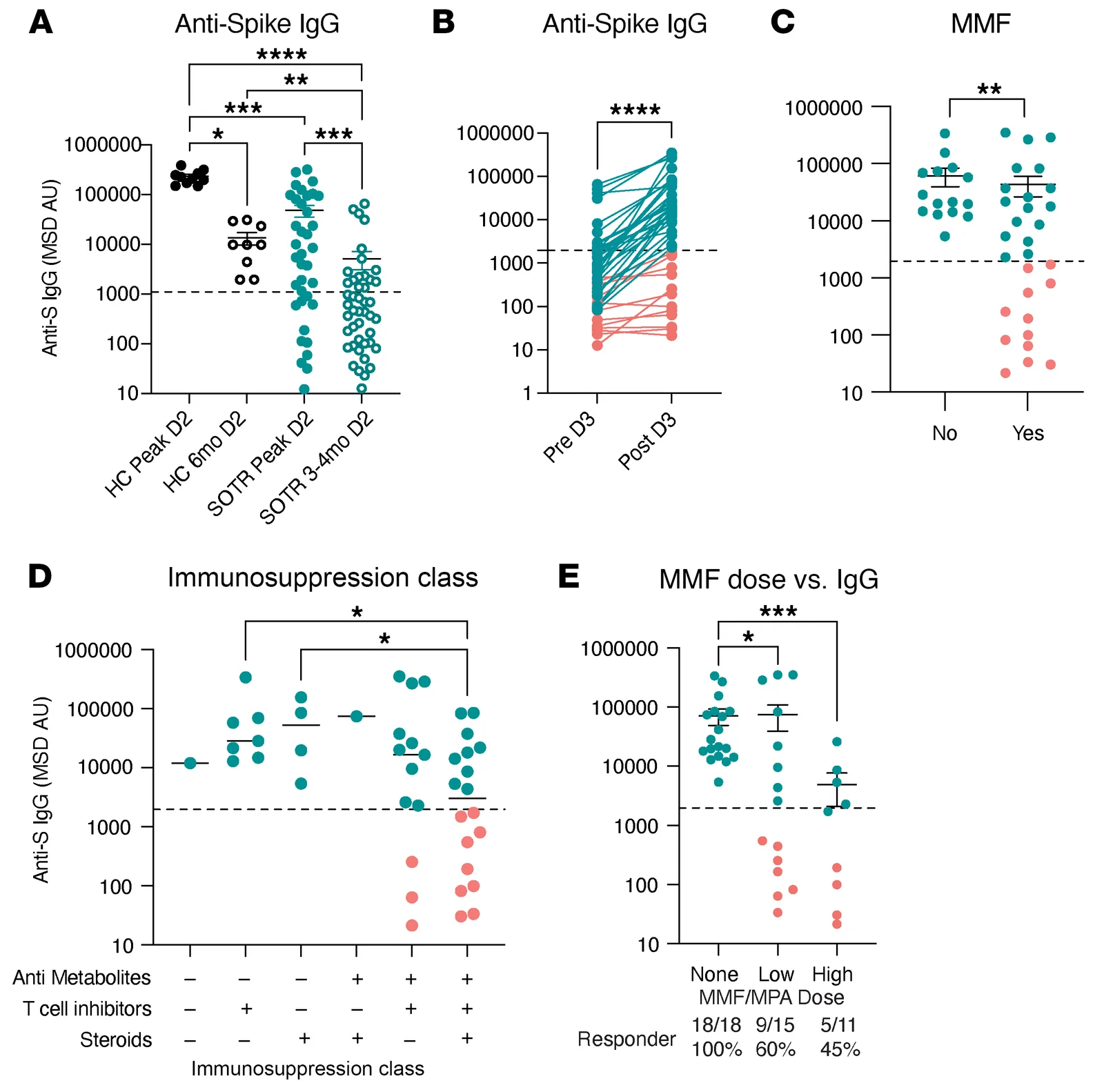
MMF is associated with a poor vaccine response. (**A**) Anti–S IgG titers in COVID-19 mRNA–vaccinated HCs (*n* = 9) at peak response 2 weeks after D2 and 6 months after D2, and SOTRs (*n* = 44) at peak response 2 weeks after D2 and 3–4 months after D2. Positive anti–S titers as determined by the manufacturer’s cutoff denoted by a dotted line. (**B**) Matched anti–S IgG titers in SOTRs before (Pre) D3 (3–4 months after D2) and 2 weeks after (Post) D3. In **B**–**E**, responders (positive IgG titers after D3, green) and nonresponders (pink) are indicated. (**C**) Anti–S IgG titers in SOTRs after D3 stratified by recipients receiving MMF or MPA. (**D**) Anti–S IgG titers in SOTRs after D3 stratified by the immunosuppressor received. (**E**) Anti–S IgG titers in SOTRs after D3 stratified by normalized daily MMF or MPA dose. Data are presented as the mean ± SEM. Significance was tested using the Kruskal-Wallis (**A**, **D**, and **E**) or Wilcoxon (**B** and **C**) test. *P < 0.05, **P < 0.01, ***P < 0.001, and ****P < 0.0001.

**Figure 2:**
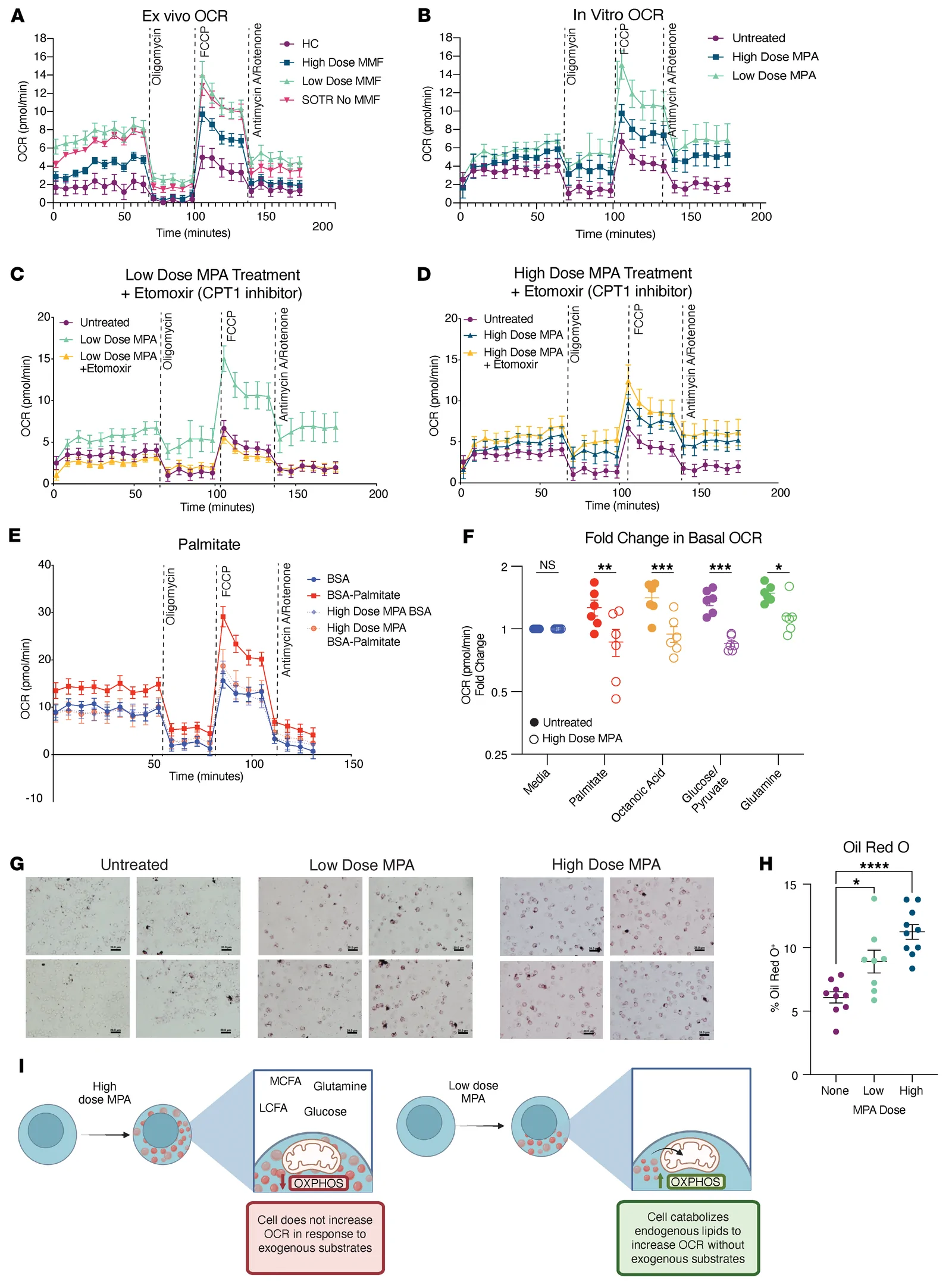
MMF and MPA directly modulate immune cell lipid metabolism. (**A**–**D**) Seahorse OCR following a mitochondrial stress test. (**A**) OCR of ex vivo PBMCs from SOTRs receiving no MMF, high-dose MMF, or low-dose MMF and HCs (*n* = 3 per group and average of 4 replicates per condition). (**B**) OCR of healthy PBMCs treated in vitro with high-dose (5 μM) or low-dose (0.05 μM) MPA for 24 hours followed by culturing in limited nutrient media for 24 hours prior to a mitochondrial stress test (*n* = 3 per group and average of 4 wells per condition). (**C** and **D**) Healthy PBMCs were prepared for mitochondrial stress test as described in **A** and exposed to etomoxir (2 μM) 30 minutes prior to the assay. (**E**) Healthy PBMCs were prepared for the mitochondrial stress test as described in **A** and exposed to BSA-palmitate (16.5 μM) or BSA 30 minutes prior to assay. (**F**) Summary of induction of the OCR at baseline following supplementation with palmitate (16.5 μM), octanoic acid (200 μM), glucose/pyruvate (10 mM/1 mM), or glutamine (2 mM) 30 minutes prior to plating. Data are normalized to media only controls from untreated to high-dose MPA–treated wells (*n* = 6 per group and average of 4 wells per condition). (**G**) Healthy PBMCs were treated in vitro with high-dose (5 μM) or low-dose (0.05 μM) MPA for 24 hours and stained with Oil Red O to measure lipid droplet accumulation. Scale bars: 20 μm. (**H**) The proportion of cells occupied by Oil Red O staining was quantified using CellProfiler; each dot represents 1 field of vision. (**I**) Proposed effects of MPA on lipid metabolism. Data are presented as the mean ± SEM. Significance tested using 2way ANOVA (**F**) or Kruskal-Wallis (**E**). *P < 0.05, **P < 0.01, ***P < 0.001, and ****P < 0.0001.

**Figure 3:**
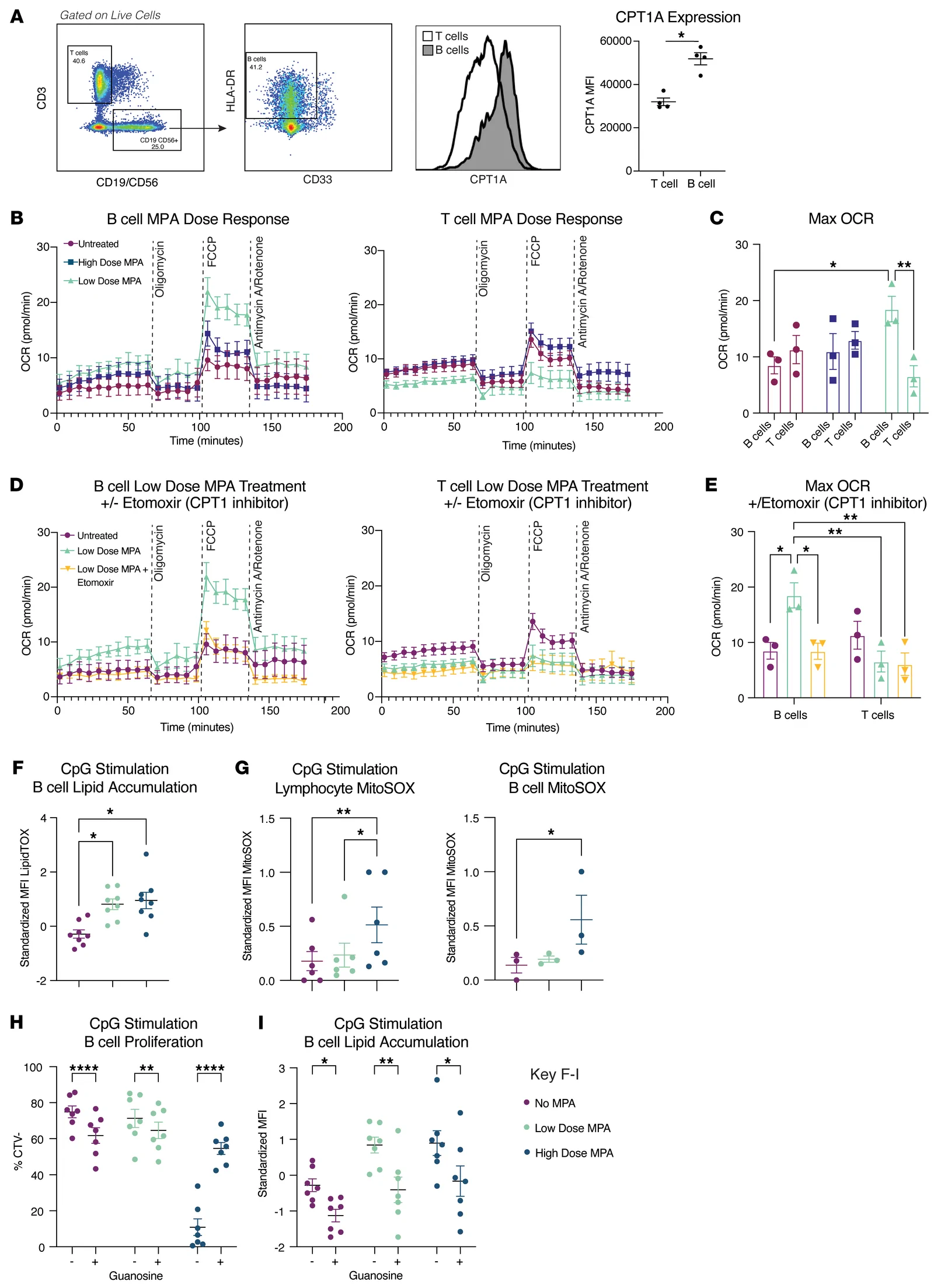
B cells demonstrate lipid accumulation and increased FAO in response to MPA. (**A**) CPT1a expression in CD3^+^ T cells and CD19^+^ B cells measured by flow cytometry, with representative gating, histograms, and quantified MFI. (**B**–**E**) Seahorse OCR following mitochondrial stress testing. (**B**) OCR of sorted B cells and T cells from HCs treated in vitro with low-dose (0.05 μM) or high-dose (5 μM) MPA for 48 hours, followed by 24 hours in limited nutrient medium before the assay (*n* = 3 per group; *n* = 4 technical replicates per condition). (**C**) Summary of the maximal (Max) OCR in sorted B cells and T cells. (**D** and **E**) OCR of sorted B cells and T cells prepared as in **B** and treated with etomoxir (2 μM) 30 minutes before the assay, with a summary of the maximal OCR shown in **E**. (**F**) B cells stimulated with CpG (2.5 μg/mL) for 6 days in the presence of low- or high-dose MPA and evaluated for total lipid content by LipidTOX staining. (**G**) MitoSOX staining of PBMCs stimulated as in **F** was done to assess mitochondrial ROS. (**H** and **I**) PBMCs were stimulated as in **F** and supplemented with guanosine (25 μM) were assessed for proliferation by CellTrace Violet (CTV) dilution and lipid accumulation by LipidTOX staining. Data are presented as the mean ± SEM. Statistical significance was assessed using the Mann-Whitney *U* test (**A**), the Friedman test (**F** and **G**), or 2-way ANOVA (**C**, **E**, **H**, and **I**). *P < 0.05, **P < 0.01, and ****P < 0.0001.

**Figure 4:**
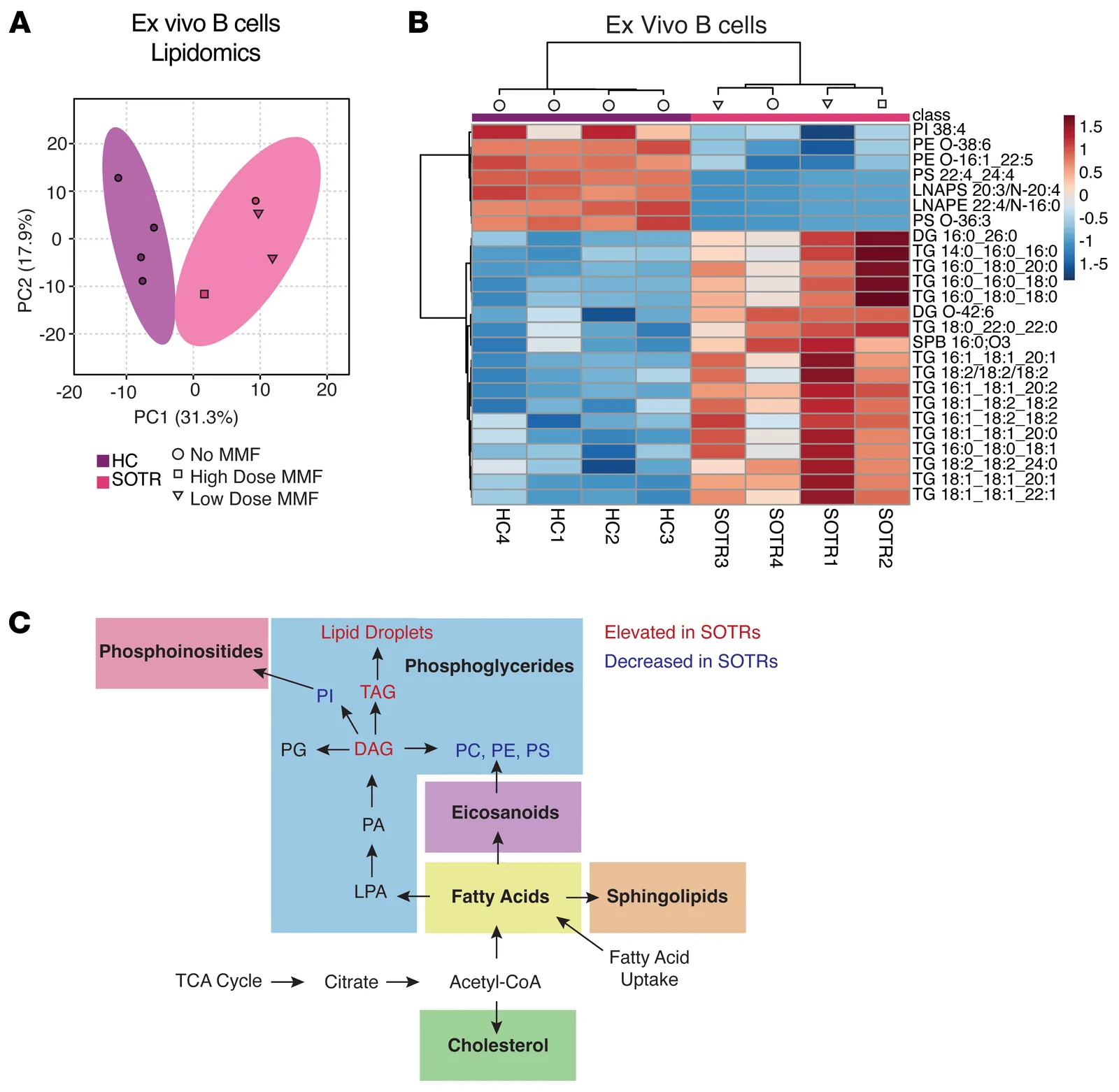
B cells from SOTRs demonstrate altered lipid profiles compared with B cells from HCs. (**A** and **B**) Ex vivo lipidomics profiling of sorted B cells from HCs and SOTRs, shown by PCA and a heatmap of the top 25 differentially expressed lipid species. (**C**) Overview of major lipid classes adapted from ref. 40 and annotated by the direction of change in SOTRs.

**Figure 5:**
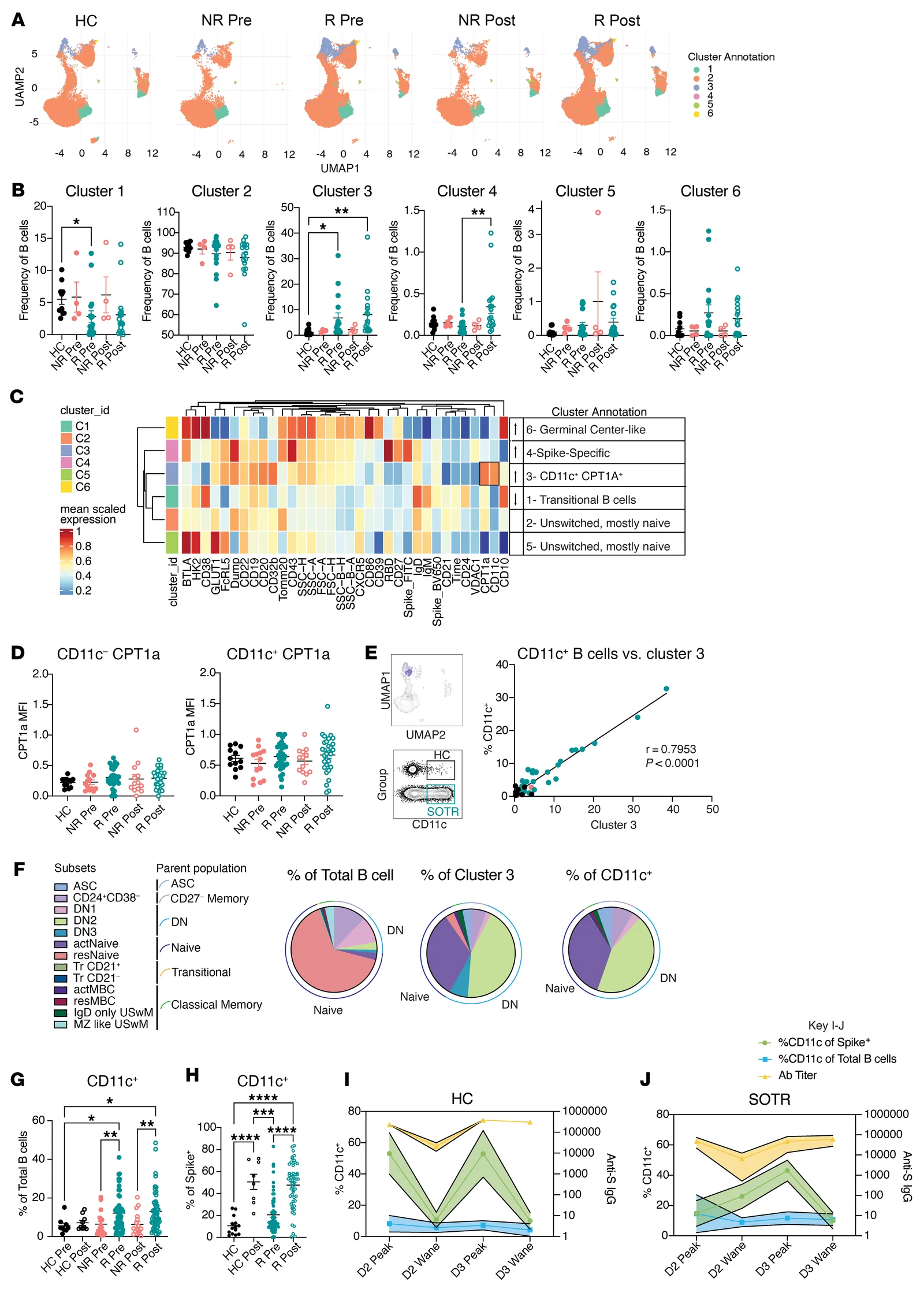
CPT1a^+^CD11c^+^ B cells are associated with vaccine responses in SOTRs. (**A**) Concatenated flow cytometric data depicted as UMAP of CD19^+^ B cells colored by cluster annotation, as determined by FlowSOM using data collected from a subset of the observational cohort before and 2 weeks after D3 (*n* = 10 HCs, *n* = 4 nonresponders [NR], *n* = 17 responders [R]). (**B**) Frequency of FlowSOM clusters segregated by response. (**C**) Heatmap depicting mean scaled expression of all markers annotated by FlowSOM cluster. (**D**) Normalized expression of CPT1a (MFI) on total CD11c^–^ or CD11c^+^ B cells. (**E**) Concatenated files demonstrating identification of cluster 3 B cells and CD11c^+^ B cells and the correlation between cells. Correlation tested using Spearman correlation. (**F**) B cells were gated as total CD19^+^ B cells, cluster 3^+^ B cells, or CD11c^+^ B cells. Pie charts depict the percentage of subsets within total (left), cluster 3 (middle), and CD11c^+^ B cells (right). Arcs depict higher-level annotations (see Supplemental Figure 7, A and B, for the gating scheme). (**G** and **H**) Frequency of CD11c^+^ B cells out of total B cells (**G**) or S-specific B cells (**H**) from combined cohorts (see Supplemental Figure 7, C–E for individual cohorts). (**I** and **J**) Additional samples were evaluated for the frequency of CD11c^+^ B cells among total B cells or S-specific B cells compared with anti–S IgG titers in HCs and SOTRs. Data are segregated by the peak response (≤30 days after D3) or waning response (>30 days after D3). Data are presented as the mean ± SEM. Significance was tested using Kruskal-Wallis test (**B**, **D**, **G**, and **H**). Correlation was tested using Spearman’s correlation (**E**). *P < 0.05, **P < 0.01, ***P < 0.001, and ****P < 0.0001.

**Figure 6:**
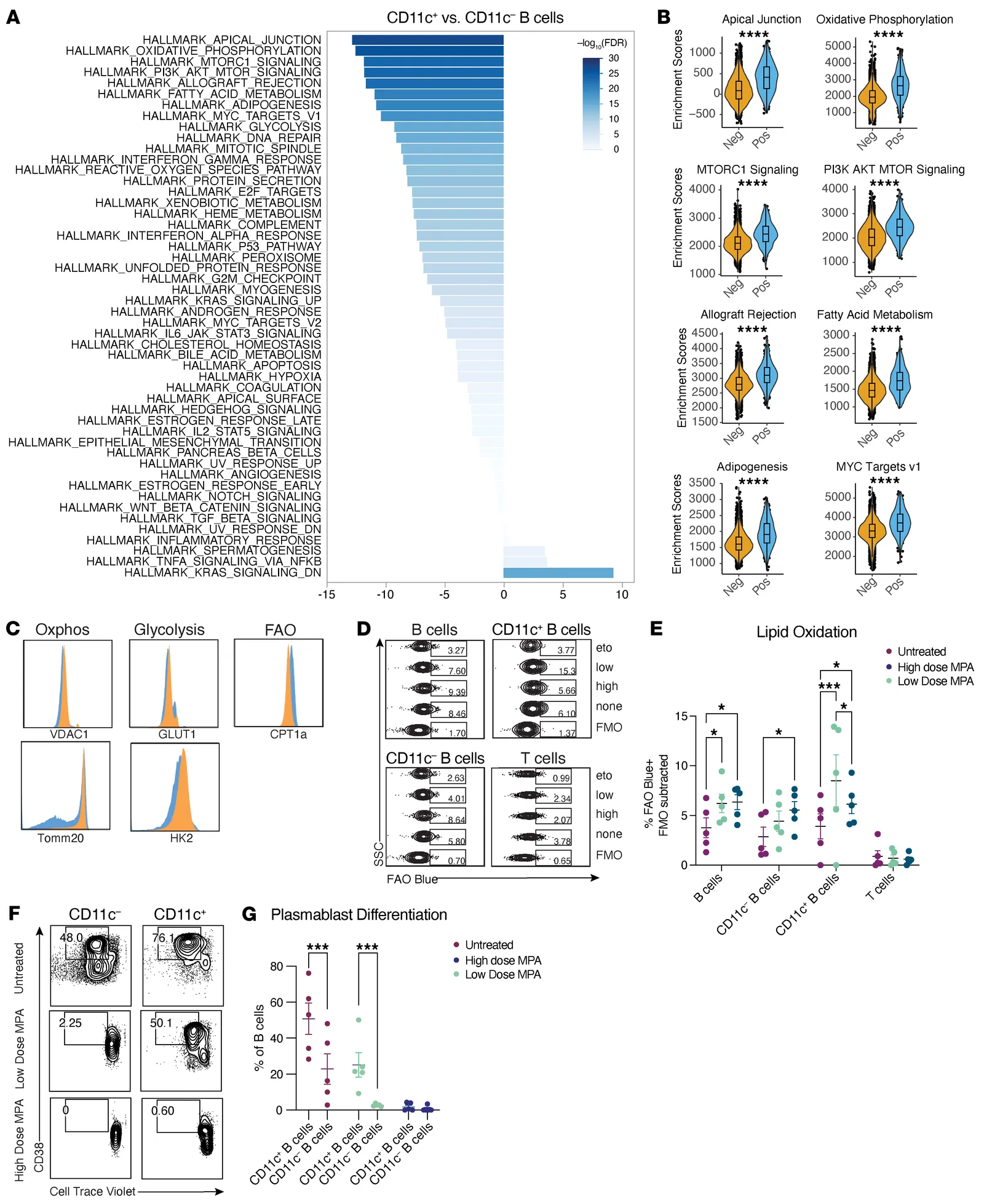
CD11c^+^ B cells show enhanced FAO capacity and maintain their ability to differentiate into plasmablasts in the presence of MPA. (**A**) scRNA-seq was performed on sorted B cells from HCs and SOTRs before and after D3. B cells (*n* = 5,548 cells) were segregated into CD11c^–^ B cells (*n* = 5,259 cells) and CD11c*^+^* B cells (*n* = 253 cells) using expression of *ITGAX*, and gene set enrichment was performed at the single-cell level using the escape platform and hallmark gene sets. Enrichment scores were tested for significant differences between CD11c^+^ and CD11c^–^ B cells and are colored according to –log_10_(FDR) corrected *P* values. (**B**) Enrichment scores for CD11c^–^ (Neg) and CD11c^+^ (Pos) B cells for the top differentially expressed gene sets. Each dot represents 1 cell. (**C**) Concatenated file of flow cytometric data from Figure 3A overlaying CD11c^+^ (blue) and CD11c^–^ (orange) B cells with expression of key metabolic proteins and enzymes denoted. (**D** and **E**) Healthy PBMCs were treated in vitro with high-dose (5 μM) or low-dose (0.05 μM) MPA for 24 hours followed by culturing in limited nutrient media for 24 hours prior to analysis with FAO-blue. Representative plots (**D**) and summary data (**E**) are shown for indicated subsets. Frequencies of FAO-blue positive cells in the fluorescence minus one (FMO) controls were subtracted for each cell type by donor. (**F** and **G**) CD11c^+^ and CD11c^–^ B cells were isolated from healthy PBMCs and stimulated with CpG (0.625 μg/mL), CD40L (0.25 μL/mL), and IL-21 (12.5 ng/mL) for 6 days in the presence of high- (5 μM) or low-dose (0.05 μM) MPA. On day 6 cells were evaluated by flow cytometry for plasmablast differentiation as assessed by proliferation (cell proliferation dye e-Fluor 450) and upregulation of CD38. Data are presented as the mean ± SEM. Enrichment scores were tested for significant enrichment using a Welch’s *t* test, and *P* values were adjusted using Benjamini-Hochberg method (**A** and **B**). Significance was tested using 2-way ANOVA (**E** and **G**). *P < 0.05, ***P < 0.001, and ****P < 0.0001.

**Figure 7:**
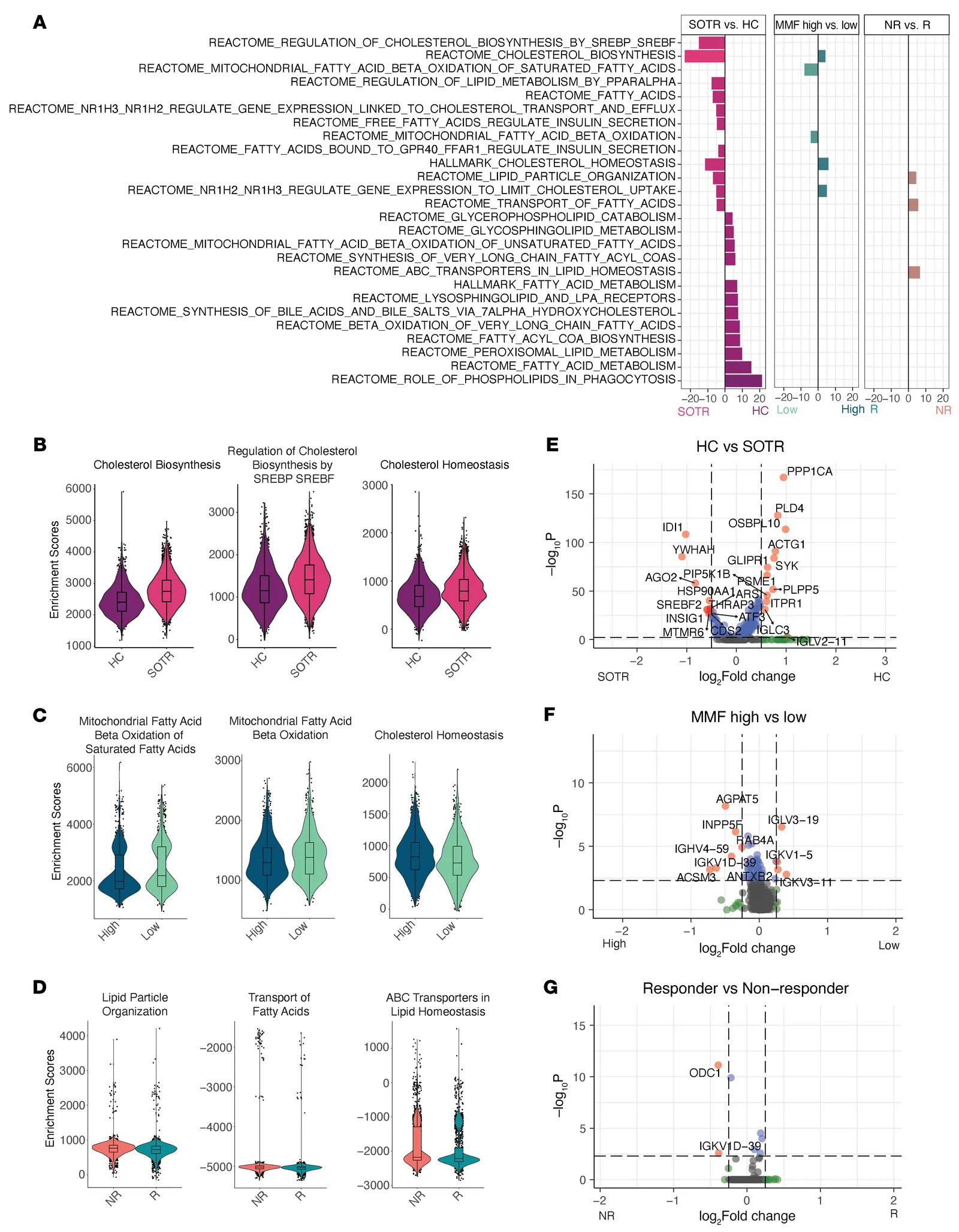
Immunosuppression induces lipid synthesis. (**A**–**G**) scRNA-seq was performed on isolated B cells from HCs and SOTRs before and after D3. Gene set enrichment was performed at the single-cell level using the escape platform and a curated set of lipid related gene sets (Supplemental Table 4). Enrichment scores were tested for significant differences between B cells (*n* = 5,549 cells) from HCs (*n* = 1,619 cells) versus SOTRs (*n* = 3,929 cells), or among SOTRs comparing high-dose MMF (*n* = 1,696 cells) versus low-dose MMF (*n* = 1,164 cells) and responders (*n* = 2,150 cells) versus nonresponders (*n* = 1,779 cells) using a Welch’s *t* test; *P* values in **A** were adjusted using the Benjamini-Hochberg method. (**A**) Gene sets with significantly different enrichment scores. (**B**–**D**) Enrichment scores for the top differentially expressed gene sets. Each dot represents 1 cell. (**E**–**G**) Volcano plot of individual genes included in lipid related gene sets. Differential expression analysis was performed using a 2-tailed Wilcoxon rank-sum test implemented in Seurat (versions 4 and 5). *P* values were adjusted for multiple hypothesis testing using Bonferroni correction based on all genes in the dataset (**E**–**G**).

**Figure 8:**
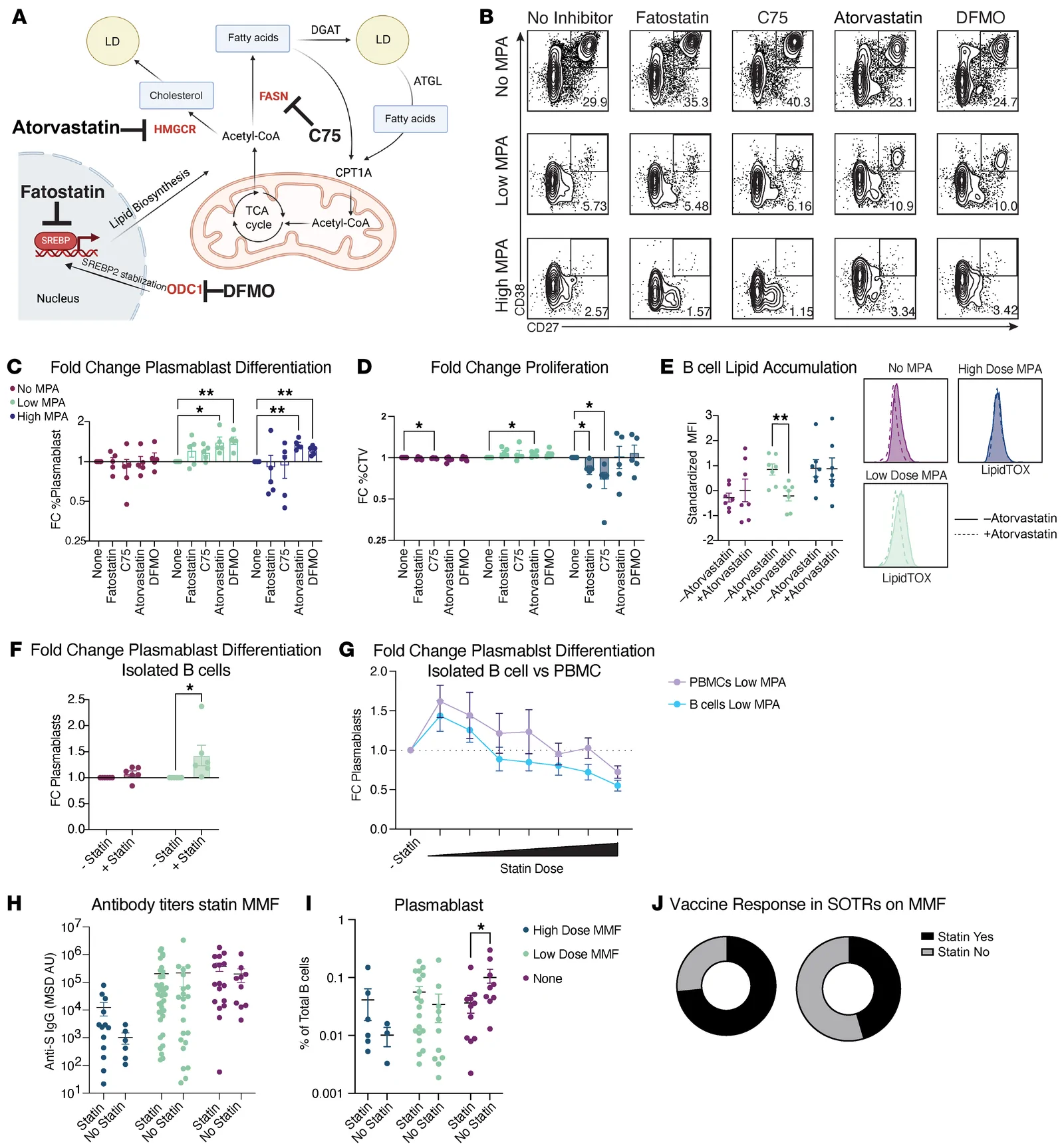
Inhibition of cholesterol synthesis improves plasmablast differentiation in the presence of MPA. (**A**) Schematic representation of the lipid synthesis inhibitors tested. (**B**–**E**) Healthy PBMCs were stimulated with CpG (2.5 μg/mL) for 6 days in the presence of high-dose (5 μM) or low-dose (0.05 μM) MPA. Cells were simultaneously supplemented with inhibitors of lipid synthesis: fatostatin (SREBP inhibitor, 0.2 μM), C75 (FA synthase inhibitor, 2 μM), atorvastatin (3-hydroxy-3-methylglutaryl-coenzyme A [HMGCoA] inhibitor, 0.02 μM), or eflornithine (also known as α-difluoromethylornithine [DFMO]) (ornithine decarboxylase 1 [ODC1] inhibitor, 0.05 μM). On day 6, cultures were evaluated by flow cytometry for plasmablast differentiation, as assessed by upregulation of CD27 and CD38 (**C**), and proliferation, as assessed by low CTV staining (**D**). On day 6, total B cells were evaluated for lipid droplet accumulation, as assessed by lipidTOX staining (**E**). (**F** and **G**) Healthy PBMCs or isolated total B cells were stimulated with CpG (2.5 μg/mL) for 6 days in the presence of no MPA or low-dose MPA (0.15 μM), as indicated and supplemented with atorvastatin (0.02 μM) (**F**) or a 2-fold dose titration of atorvastatin ranging from 32 μM to 0.007 μM (**G**). (**H**–**J**) Participants in NCT04969263 were screened for co-medications including statins. Anti–S IgG titers (**H**) and total plasmablast frequencies (**I**) were evaluated 2 weeks after D3, and participants were segregated according to high-dose MMF or MPA, low-dose MMF or MPA, or no MMF or MPA. (**J**) Participants in NCT04969263 taking MMF or MPA were segregated for responders and nonresponders and evaluated for the presence of statins as a comedication. Data are presented as the mean ± SEM. Significance was tested using 2-way ANOVA (**C**–**E**, **H**, and **I**). *P < 0.05 and **P < 0.01.

**Figure 9:**
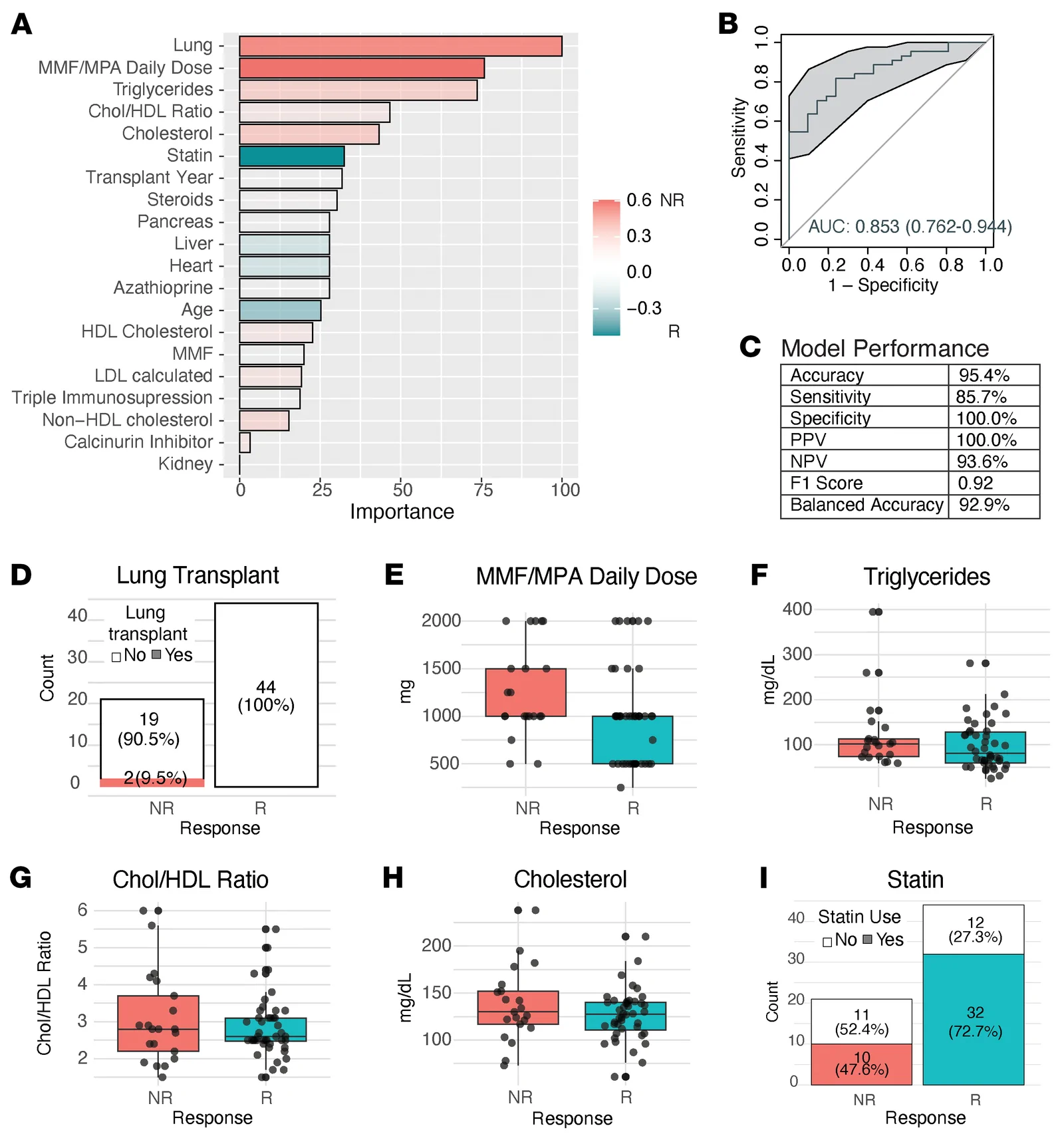
Machine learning identifies measurements of lipid metabolism associated with vaccine responses. (**A**) Feature importance from a random forest model distinguishing responders (*n* = 44) from nonresponders (*n* = 21) among SOTRs taking MMF or MPA. Importance value shown is the mean decreasing accuracy. The color of the bars was calculated as the change in mean value between responders and nonresponders to represent enrichment in either category. (**B**) ROC analysis using the variables shown in **A** to estimate the accuracy of predicting the response to a third vaccine dose. The AUC was calculated. (**C**) Additional model performance metrics demonstrating strong classification performance, with 95.4% accuracy, 85.7% sensitivity, 100% specificity, 100% positive predictive value (PPV), 93.6% negative predictive value (NPV), an F1 score of 0.92, and 92.9% balanced accuracy. (**D**–**I**) Plots of individual top variables as indicated. Chol, cholesterol.

## Version 1 09/17/2026

In-Press Preview

## Version 2 11/16/2026

Electronic publication

## References

[1] Martinson ML, Lapham J (2024). Prevalence of immunosuppression among US adults. JAMA.

[2] Lopez A (2017). Vaccination recommendations for the adult immunosuppressed patient: a systematic review and comprehensive field synopsis. J Autoimmun.

[3] Haidar G (2022). Prospective evaluation of coronavirus disease 2019 (COVID-19) vaccine responses across a broad spectrum of immunocompromising conditions: the COVID-19 vaccination in the immunocompromised study (COVICS). Clin Infect Dis.

[4] Qin CX (2021). Risk of breakthrough SARS-CoV-2 infections in adult transplant recipients. Transplantation.

[5] Solera JT (2024). Longitudinal outcomes of COVID-19 in solid organ transplant recipients from 2020 to 2023. Am J Transplant.

[6] Sun J (2022). Association between immune dysfunction and COVID-19 breakthrough infection after SARS-CoV-2 vaccination in the US. JAMA Intern Med.

[7] Karaba AH (2022). A third dose of SARS-CoV-2 vaccine increases neutralizing antibodies against variants of concern in solid organ transplant recipients. Am J Transplant.

[8] Charmetant X (2022). Infection or a third dose of mRNA vaccine elicits neutralizing antibody responses against SARS-CoV-2 in kidney transplant recipients. Sci Transl Med.

[9] Werbel WA (2021). Safety and immunogenicity of a third dose of SARS-CoV-2 vaccine in solid organ transplant recipients: a case series. Ann Intern Med.

[10] Hall VG (2021). Randomized trial of a third dose of mRNA-1273 vaccine in transplant recipients. N Engl J Med.

[11] Falsey AR (2021). SARS-CoV-2 neutralization with BNT162b2 vaccine dose 3. N Engl J Med.

[12] Müller TR (2023). Additive effects of booster mRNA vaccination and SARS-CoV-2 Omicron infection on T cell immunity across immunocompromised states. Sci Transl Med.

[13] Patel CH (2019). Targeting metabolism to regulate immune responses in autoimmunity and cancer. Nat Rev Drug Discov.

[14] Buck MD (2015). T cell metabolism drives immunity. J Exp Med.

[15] O’Neill LAJ, Pearce EJ (2015). Immunometabolism governs dendritic cell and macrophage function. J Exp Med.

[16] Reischig T (2022). Insufficient response to mRNA SARS-CoV-2 vaccine and high incidence of severe COVID-19 in kidney transplant recipients during pandemic. Am J Transplant.

[17] Lentine KL (2024). OPTN/SRTR 2022 annual data report: kidney. Am J Transplant.

[18] Bhat R (2023). Perspectives on mycophenolate mofetil in the management of autoimmunity. Clin Rev Allergy Immunol.

[19] Allison AC, Eugui EM (2000). Mycophenolate mofetil and its mechanisms of action. Immunopharmacology.

[20] Boyarsky BJ (2021). Antibody response to 2-dose SARS-CoV-2 mRNA vaccine series in solid organ transplant recipients. JAMA.

[21] Kantauskaite M (2022). Intensity of mycophenolate mofetil treatment is associated with an impaired immune response to SARS-CoV-2 vaccination in kidney transplant recipients. Am J Transplant.

[22] Mitchell J (2022). Effect of mycophenolate mofetil dosing on antibody response to SARS-CoV-2 vaccination in heart and lung transplant recipients. Transplantation.

[23] Fatly ZA (2024). Mycophenolate mofetil hampers antibody responses to a broad range of vaccinations in kidney transplant recipients: results from a randomized controlled study. J Infect.

[24] Lupu M (2006). Pharmacokinetics of oral mycophenolate mofetil in dog: bioavailability studies and the impact of antibiotic therapy. Biol Blood Marrow Transplant.

[25] Eugui EM (1991). Lymphocyte-selective cytostatic and immunosuppressive effects of mycophenolic acid in vitro: role of deoxyguanosine nucleotide depletion. Scand J Immunol.

[26] Guzera M (2016). In vitro influence of mycophenolic acid on selected parameters of stimulated peripheral canine lymphocytes. PLoS One.

[27] Armstrong VW (2005). Pharmacokinetics and bioavailability of mycophenolic acid after intravenous administration and oral administration of mycophenolate mofetil to heart transplant recipients. Ther Drug Monit.

[28] Chmielnicka K (2022). Dyslipidemia in renal transplant recipients. Transplantology.

[29] Hricik DE (1994). Posttransplant hyperlipidemia: the treatment dilemma. Am J Kidney Dis.

[30] Warden BA, Duell PB (2019). Management of dyslipidemia in adult solid organ transplant recipients. J Clin Lipidol.

[31] Li S (2017). Metabolic phenotypes of response to vaccination in humans. Cell.

[32] Luo W (2023). SREBP signaling is essential for effective B cell responses. Nat Immunol.

[33] [no authors listed] (2023). Lipid homeostasis mediated by cholesterol synthesis supports B cell responses to vaccination. Lipid homeostasis mediated by cholesterol synthesis supports B cell responses to vaccination. Nat Immunol.

[34] Raud B (2018). Etomoxir actions on regulatory and memory T cells are independent of Cpt1a-mediated fatty acid oxidation. Cell Metab.

[35] Yao C-H (2018). Identifying off-target effects of etomoxir reveals that carnitine palmitoyltransferase I is essential for cancer cell proliferation independent of β-oxidation. PLoS Biol.

[36] Divakaruni AS (2018). Etomoxir inhibits macrophage polarization by disrupting CoA homeostasis. Cell Metab.

[37] Nomura M (2019). Macrophage fatty acid oxidation inhibits atherosclerosis progression. J Mol Cell Cardiol.

[38] Malandrino MI (2015). Enhanced fatty acid oxidation in adipocytes and macrophages reduces lipid-induced triglyceride accumulation and inflammation. Am J Physiol Endocrinol Metab.

[39] Olzmann JA, Carvalho P (2019). Dynamics and functions of lipid droplets. Nat Rev Mol Cell Biol.

[40] Baenke F (2013). Hooked on fat: the role of lipid synthesis in cancer metabolism and tumour development. Dis Model Mech.

[41] Quintelier K (2021). Analyzing high-dimensional cytometry data using FlowSOM. Nat Protoc.

[42] Karnell JL (2017). Role of CD11c^+^ T-bet^+^ B cells in human health and disease. Cell Immunol.

[43] Gao X, Cockburn IA (2022). The development and function of CD11c^+^ atypical B cells - insights from single cell analysis. Front Immunol.

[44] Sutton HJ (2021). Atypical B cells are part of an alternative lineage of B cells that participates in responses to vaccination and infection in humans. Cell Rep.

[45] Woodruff MC (2020). Extrafollicular B cell responses correlate with neutralizing antibodies and morbidity in COVID-19. Nat Immunol.

[46] Faliti CE (2024). Disease-associated B cells and immune endotypes shape adaptive immune responses to SARS-CoV-2 mRNA vaccination in human SLE. Nat Immunol.

[47] Lopes de Assis F (2023). Tracking B cell responses to the SARS-CoV-2 mRNA-1273 vaccine. Cell Rep.

[48] Steuten J (2023). Distinct dynamics of antigen-specific induction and differentiation of different CD11c^+^Tbet^+^ B-cell subsets. J Allergy Clin Immunol.

[49] Sanz I (2019). Challenges and opportunities for consistent classification of human B cell and plasma cell populations. Front Immunol.

[50] Eisenbarth SC (2025). A roadmap for defining “extrafollicular” B cell responses. Immunity.

[51] Golinski M-L (2020). CD11c^+^ B cells are mainly memory cells, precursors of antibody secreting cells in healthy donors. Front Immunol.

[52] Wang S (2018). IL-21 drives expansion and plasma cell differentiation of autoreactive CD11chiT-bet^+^ B cells in SLE. Nat Commun.

[53] Jenks SA (2018). Distinct effector B cells induced by unregulated Toll-like receptor 7 contribute to pathogenic responses in systemic lupus erythematosus. Immunity.

[54] O’Sullivan D (2014). Memory CD8(^+^) T cells use cell-intrinsic lipolysis to support the metabolic programming necessary for development. Immunity.

[55] Horton JD (1998). Activation of cholesterol synthesis in preference to fatty acid synthesis in liver and adipose tissue of transgenic mice overproducing sterol regulatory element-binding protein-2. J Clin Invest.

[56] Horton JD (2002). SREBPs: activators of the complete program of cholesterol and fatty acid synthesis in the liver. J Clin Invest.

[57] Firpo MR (2023). Polyamines and eIF5A hypusination facilitate SREBP2 synthesis and cholesterol production leading to enhanced enterovirus attachment and infection. PLoS Pathog.

[58] Cardoso D, Perucha E (2021). Cholesterol metabolism: a new molecular switch to control inflammation. Clin Sci (Lond).

[59] Goicoechea L (2023). Mitochondrial cholesterol: metabolism and impact on redox biology and disease. Redox Biol.

[60] Shimabukuro-Vornhagen A (2014). Inhibition of protein geranylgeranylation specifically interferes with CD40-dependent B cell activation, resulting in a reduced capacity to induce T cell immunity. J Immunol.

[61] Li J (2022). Factors associated with COVID-19 vaccine response in transplant recipients: a systematic review and meta-analysis. Transplantation.

[62] Manothummetha K (2022). Immunogenicity and risk factors associated with poor humoral immune response of SARS-CoV-2 vaccines in recipients of solid organ transplant: a systematic review and meta-analysis. JAMA Netw Open.

[63] Rodrigo E (2012). ImmuKnow as a diagnostic tool for predicting infection and acute rejection in adult liver transplant recipients: a systematic review and meta-analysis. Liver Transpl.

[64] Nash A (2020). Effects of immunosuppressive medications on mitochondrial function. J Surg Res.

[65] Heischmann S (2017). The immunosuppressant mycophenolic acid alters nucleotide and lipid metabolism in an intestinal cell model. Sci Rep.

[66] Zheng Z-H (2011). Mycophenolic acid induces adipocyte-like differentiation and reversal of malignancy of breast cancer cells partly through PPARγ. Eur J Pharmacol.

[67] Voloshyna I (2019). Macrophage lipid accumulation in the presence of immunosuppressive drugs mycophenolate mofetil and cyclosporin A. Inflamm Res.

[68] Cohn RG (1999). Mycophenolic acid increases apoptosis, lysosomes and lipid droplets in human lymphoid and monocytic cell lines. Transplantation.

[69] Chandel NS (2021). NADPH-the forgotten reducing equivalent. Cold Spring Harb Perspect Biol.

[70] Hardie DG, Pan DA (2002). Regulation of fatty acid synthesis and oxidation by the AMP-activated protein kinase. Biochem Soc Trans.

[71] Boothby M, Rickert RC (2017). Metabolic regulation of the immune humoral response. Immunity.

[72] Raza IGA, Clarke AJ (2021). B cell metabolism and autophagy in autoimmunity. Front Immunol.

[73] Weisel FJ (2020). Germinal center B cells selectively oxidize fatty acids for energy while conducting minimal glycolysis. Nat Immunol.

[74] Sharma R (2023). Distinct metabolic requirements regulate B cell activation and germinal center responses. Nat Immunol.

[75] Brookens SK (2024). Plasma cell differentiation, antibody quality, and initial germinal center B cell population depend on glucose influx rate. J Immunol.

[76] Li M (2024). CPT2-mediated fatty acid oxidation is dispensable for humoral immunity. J Immunol.

[77] Black S (2016). Influence of statins on influenza vaccine response in elderly individuals. J Infect Dis.

[78] McLean HQ (2016). Effect of statin use on influenza vaccine effectiveness. J Infect Dis.

[79] Omer SB (2016). Impact of statins on influenza vaccine effectiveness against medically attended acute respiratory illness. J Infect Dis.

[80] Hao Y (2011). A B-cell subset uniquely responsive to innate stimuli accumulates in aged mice. Blood.

[81] Rubtsov AV (2011). Toll-like receptor 7 (TLR7)-driven accumulation of a novel CD11c^+^ B-cell population is important for the development of autoimmunity. Blood.

[82] Zhang W (2019). Excessive CD11c^+^Tbet^+^ B cells promote aberrant TFH differentiation and affinity-based germinal center selection in lupus. Proc Natl Acad Sci U S A.

[83] Sundling C (2019). B cell profiling in malaria reveals expansion and remodeling of CD11c^+^ B cell subsets. JCI Insight.

[84] Knox JJ (2017). T-bet^+^ B cells are induced by human viral infections and dominate the HIV gp140 response. JCI Insight.

[85] Glass DR (2020). An integrated multi-omic single-cell atlas of human B cell identity. Immunity.

