# Supplementary material for: Balancing lipid synthesis and oxidation promotes B cell response to vaccination during immunosuppression
Source: J Clin Invest. 2026 Sep 17;136(22):e205170. doi: 10.1172/JCI205170 (PMC13767638; doi:10.1172/JCI205170)
Supplement: Supplementary file 1 [file jci-136-205170-s142.pdf]

Supplementary Materials

Balancing lipid synthesis and oxidation promotes B cell response to vaccination during immunosuppression

Thompson et al

Materials and Methods

Blood Processing and In Vitro PBMC Isolation

Blood was collected in acid citrate dextrose or heparin tubes. Plasma was isolated by centrifugation and stored at –80°C until antibody measurement. PBMCs were isolated within 24 hours of blood collection via density gradient centrifugation over Ficoll-Paque at 400*g* for 30 min without brake, washed with RPMI-1640, and cryopreserved in liquid nitrogen. In vitro assays utilized leukopaks and whole blood from deidentified healthy donors processed using the same standard density gradient centrifugation protocol.

SARS-CoV-2 Antibody Titer Measurements

Plasma anti-N, anti-RBD, and anti-S IgG were measured using the multiplex chemiluminescent Meso Scale Diagnostics V-PLEX COVID-19 Respiratory Panel 3 Kit according to the manufacturer's protocol at a 1:5000 dilution. Endpoint titers for spike-specific IgM, IgA, and IgG subclasses were determined by indirect ELISA as previously described. Briefly, 96-well ELISA plates (Immulon 4HBX, Thermo Fisher Scientific) were coated with recombinant SARS-CoV-2 spike protein (2 µg/mL, 50 µl/well) overnight at 4°C. Plates were washed, blocked with 3% nonfat milk for 1 h at room temperature (RT), and incubated with 3-fold serially diluted plasma (starting at 1:20) for 2 h at RT. Horseradish peroxidase (HRP)-conjugated secondary antibodies were added for 1 h at RT: IgM (Invitrogen, Cat# A18835), IgA (Invitrogen, Cat# A18787), IgG (Invitrogen, Cat# A18823), IgG1 (SouthernBiotech, Cat# 9054-05), IgG2 (SouthernBiotech, Cat# 9060-05), IgG3 (SouthernBiotech, Cat# 9210-05), and IgG4 (SouthernBiotech, Cat# 9200-05). Reactions were developed using Sigmafast OPD solution (MilliporeSigma) for 10 min, stopped with 3 M hydrochloric acid (50 µl/well), and read at 490 nm. Endpoint titers were calculated using cutoff values defined as the plate-specific average plus 3 times the standard deviation of negative control samples.

Ex Vivo Spectral Flow Cytometry and Probe Preparation

PBMCs were used for phenotypic and metabolic assessment. Antigen-specific B cell probes were generated from biotinylated full-length spike and RBD proteins mixed at a 4:1 protein-to-fluorochrome molar ratio with streptavidin-FITC, streptavidin-BV650 (for spike), and streptavidin-PE (for RBD), calculating ratios based strictly on protein monomer and streptavidin molecular weights. Cells were washed in PBS and stained with BioLegend Live/Dead Zombie NIR Fixable Viability Dye and BD Fc Block for 10 min at RT. Surface staining was performed in 20% BD Horizon Brilliant Stain Buffer in PBS with the antibody cocktail and probes for 20 min at RT. Cells were fixed and permeabilized using the eBioscience FoxP3/Transcription Factor Staining Kit for 20 min at RT, followed by intracellular staining in 1× Permeabilization/Wash buffer for 20 min at RT. Cells were resuspended in 1% paraformaldehyde and acquired on a 4-laser Cytek Aurora spectral flow cytometer. Detailed

antibody panel configuration including clones, fluorochromes, manufacturers, and dilutions is provided in Supplemental Table S5.

High-Dimensional Flow Cytometry Analysis

FCS files were analyzed using FlowJo software (v10.6.2; manual gating strategies are detailed in Figs. S1 and S7). For unsupervised clustering, CD19+CD20+ B cell populations were exported to R (version 4.0.1). Unsupervised clustering was executed using the FlowSOM algorithm and visualized using Uniform Manifold Approximation and Projection (UMAP). Differential abundance testing was carried out using the diffcyt framework and a modified version of the CATALYST workflow (1, 2).

Extracellular Flux (Seahorse) Assays

Healthy PBMCs were rested and cultured for 24 h in complete media (RPMI-1640, 10% FBS, 1M HEPES, 10 mg/mL nonessential amino acids, and 5×10^−2 M 2-mercaptoethanol) containing low-dose mycophenolic acid (MPA; 0.05 μM), high-dose MPA (5 μM), or vehicle control. Media was replaced with nutrient-limited Seahorse XF RPMI (Agilent, Cat# 103576-100) containing 1% FBS, 0.5 mM glucose, 1 mM L-glutamine, and 0.5 mM L-carnitine (Sigma-Aldrich, Cat# C0158) for an additional 24 h without MPA. On Day 2, 150,000 cells/well were seeded onto Poly-D-lysine–coated (50 μg/mL; Sigma-Aldrich, Cat# P0899) Seahorse XF96 Cell Culture Microplates in Seahorse assay medium (XF RPMI supplemented with 2 mM glucose and 0.5 mM L-carnitine). In parallel experiments, PBMCs from SOTR patients receiving clinical regimens of high-dose mycophenolate mofetil (MMF), low-dose MMF, or no MMF were thawed and immediately plated in Seahorse assay medium. Where indicated, cells were treated 30 min prior to the assay with etomoxir (2 μM; Tocris Bioscience, Cat# 4539/10), BSA-palmitate or BSA control (16.5 μM; Cayman Chemical, Cat# 29558 / 29556), octanoic acid (200 μM; Sigma-Aldrich, Cat# C2875), glutamine (2 mM; Corning, Cat# 25-005-C), or a combination of glucose (10 mM; Sigma-Aldrich, Cat# G7528) and pyruvate (2 mM; Thermo Fisher, Cat# 11360070). Oxygen consumption rates (OCR) were measured on an XF96 Extracellular Flux Analyzer (Agilent Technologies) under sequential injections of oligomycin (1 μM), FCCP (1.5 μM), and a mixture of rotenone (2 μM) and antimycin A (1 μM). Measurements were used to derive basal respiration, ATP-linked respiration, maximal respiration, non-mitochondrial respiration, and spare respiratory capacity according to standard formulas.

In Vitro T and B Cell Proliferation Titrations

PBMCs were labeled with CellTrace Violet (CTV; Thermo Fisher, Cat# C34557) and cultured at 1×10^6 cells/mL in complete media for 6 days with anti-CD3/CD28 stimulating antibodies (0.1 mg/mL; Miltenyi Biotec, Cat# 11161D) in the presence of 0.05 μM MPA, 5 μM MPA, or vehicle. For isolated B cell assays, pan-B cells were isolated by negative magnetic selection (Miltenyi Biotec, Cat# 130-101-638) and cultured at 50,000 cells/well in flat-bottom 96-well plates. B cells were stimulated for 6 days with 2.5 μg/mL CpG oligodeoxynucleotide (Invivogen, Cat# tlrl-2006-1), 2.5 μg/mL F(ab')2 anti-human IgM/IgA/IgG (Jackson ImmunoResearch, Cat# 109-006-064), 1 μg/mL CD40L (R&D Systems, Cat# 6420-CL), and 5 U/mL IL-2 (R&D Systems, Cat# 202-IL-050/CF) under indicated MPA treatments. Proliferation was evaluated on Day 6 by flow cytometry using Zombie NIR viability dye, anti-CD3 (BUV661, BD Biosciences, Cat# 612964), and anti-CD19 (APC-Cy7, BioLegend, Cat# 363010). Note: Batch-to-batch

variation in fetal bovine serum (FBS) lots requires empirical titration of the low-dose MPA condition between 0.05 μM and 0.15 μM to achieve optimal partial antiproliferative kinetics.

Lipid Accumulation and Metabolic Function Assays
For Oil Red O imaging, PBMCs were rested for 3 h, cultured for 48 h with or without MPA, and seeded at 200,000 cells/slide onto Poly-D-lysine–coated glass slides. Slides were stained using an Oil Red O Kit (MilliporeSigma, Cat# MAK194) and mounted with glycerol gelatin aqueous medium (MilliporeSigma, Cat# GG1). Brightfield images were acquired on an Olympus BX51 microscope using a UIS2 40X/0.75 objective and a DP10 color CCD camera. Percent positive lipid staining area was quantified using CellProfiler software. For flow cytometric lipid profiling, cells stimulated for 6 days with CpG were incubated with LipidTOX Deep Red Neutral Lipid Stain (Invitrogen, 1:1000) and BODIPY-C16 (Invitrogen, 20 nM) for 30 min at 37°C. For glucose and fatty acid uptake, cells were incubated with 2-NBDG (Invitrogen) or FAO-Blue (20 nM; DiagnoCine, Cat# FNK-FDV-0033) for 30 min at 37°C. Mitochondrial membrane potential and reactive oxygen species were measured by staining unfixed cells with TMRE (Invitrogen, 0.4 μM) or MitoSOX Red (Invitrogen, 5 μM) for 30 min at 37°C, followed by immediate acquisition on a Cytek Northern Lights cytometer. To normalize batch effects across lipid staining experiments, raw mean fluorescence intensities (MFI) were transformed into Z-scores using JMP Student Version 18 according to the formula: Z = (Variable − Batch Mean) / Batch SD.

Metabolite and Lipid LC-MS Profiling
Pan-B cells were isolated by negative magnetic selection (Miltenyi Biotec, Cat# 130-101-638) from healthy and SOTR PBMCs. Total polar metabolites and lipids were extracted using a standardized methanol/methyl tert-butyl ether (MTBE)/water phase-separation protocol. The upper lipid phase and lower polar metabolite phase were isolated, dried under nitrogen gas, and stored at –80°C. Extracts were analyzed using a Vanquish UHPLC system coupled to a Q Exactive HF-X mass spectrometer (Thermo Fisher Scientific) equipped with an electrospray ionization (ESI) source running Xcalibur software. Chromatographic separation was performed using HILIC for polar metabolites and a reversed-phase method for lipids. Spectral data processing and analyte identification were performed using Compound Discoverer 3.3 against an in-house retention-time library and the LIPID MAPS Structure Database.

Single-Cell Sample Preparation and Sorting
PBMCs were obtained from solid organ transplant recipient (SOTR) vaccine non-responders (n=10), SOTR responders (n=10), and healthy controls (HC; n=4) before and after receiving a third dose of a COVID-19 mRNA vaccine. Following thawing, pan-B cells were sorted from PBMCs (Miltenyi Biotec, Cat# 130-101-638) and then stained with antibodies against CD3, CD14, and CD19, along with fluorescently labeled SARS-CoV-2 spike protein probes to identify antigen-specific cells as previously described above.

For sample multiplexing, cell hashing was performed using MULTI-seq lipid-modified oligos. Briefly, cells were washed twice with phosphate-buffered saline (PBS) and incubated on ice for 5 minutes with 200 μL of Anchor-Barcode solution (200 nM). Subsequently, 20 μL of Co-Anchor solution (200 nM) was added, and the incubation continued ice for an additional 5 minutes. Labeled cells were washed twice with ice-cold PBS containing 1% bovine serum

albumin (BSA), then pooled into a single FACS tube. Spike-reactive and non-reactive B cell fractions were sorted using a BD FACSMelody™ Cell Sorter at the Johns Hopkins Blomberg School of Public Health BD Flow Cytometry Core.

Single-Cell RNA Library Preparation and Sequencing
Approximately 10,000 spike-nonreactive and 2,000 spike-reactive B cells were processed for single-cell RNA sequencing (scRNA-seq) using the Chromium Next GEM Single Cell 3′ v3.1 kit (10x Genomics, PN-1000128). Both the gene expression (GEX) and MULTI-seq libraries were prepared according to the manufacturer's protocols and sequenced on an Illumina NovaSeq 6000 platform. The target sequencing depth was 50,000 reads/cell for the GEX library and 5,000 reads/cell for the MULTI-seq library.

Single-Cell Demultiplexing and Analysis
deMULTIplex (https://github.com/chris-mcginnis-ucsf/MULTI-seq) an R package was used for analyzing the MULTI-seq data. Cells lacking barcode tags or labeled with multiple barcodes were classified as background droplets or doublets and removed from downstream analysis.

These data were then imported into Seurat package (v3.1) for subsequent analysis. Cells were filtered to remove low-quality cells based on gene detection and mitochondrial content, retaining cells with 200 to 3000 detected genes and <10% mitochondrial gene expression. To achieve this, we called subset function while using nFeature_RNA and percent.mt. Gene expression values were normalized using a global-scaling log-normalization method. This is implemented by NormalizeData. 2,000 highly variable genes were identified using the variance-stabilizing transformation method (FindVariableFeatures, selection.method = "vst"). The data were subsequently scaled (ScaleData) prior to dimensionality reduction. Principal component analysis was performed (RunPCA) using the variable features, and the first 15 principal components were retained.

To integrate data generated from multiple donors and experimental conditions, we applied an anchor-based data integration strategy using the FindIntegrationAnchors and IntegrateData functions in Seurat, based on selected 2,000 high variable genes. This approach aligns shared cell states across datasets while reducing donor-specific technical variability. All downstream visualization and clustering were performed in the integrated expression space. The integration framework effectively controls batch effects in downstream analyses.

Single cell gene set enrichment analysis (GSEA) was performed using the Escape package (v1.5.1). Differences in CD11c+ and CD11- B cells were evaluated using Hallmark gene sets. Differences in total B cells were evaluated using a custom list of gene sets related to lipid metabolism (Table S4). Significance was tested within the Escape package using the getSignificance() command. All significant values were FDR corrected. To determine somatic mutation rates of the *IGHV* gene, sequences were submitted to IMGT High-VQUEST for comparison to germline *IGHV* gene sequences. Because somatic mutation rates in the populations were not normally distributed, as determined by the Shapiro-Wilk test, they were compared by the Wilcoxon signed-rank test.

Targeted In Vitro Pharmacological Inhibition Assays
To assess B cell and plasmablast differentiation kinetics, isolated B cells or total PBMCs were stimulated with 2.5 μg/mL CpG in complete media for 6 days. Where indicated, cultures were

treated with specific pathway inhibitors: fatostatin (SREBP inhibitor; 0.2 μM), C75 (FASN inhibitor; 2 μM), atorvastatin (HMGCR inhibitor; 0.02 μM), or DFMO (ODC1 inhibitor; 0.05 μM), with or without metabolic rescue via 25 μM guanosine (MilliporeSigma, Cat# G6752-1G). On Day 6, cells were stained with antibodies against CD19, CD11c, CD27, CD20, CD38, CD21, and IgD (see Table S2 for clones and manufacturers) and evaluated on a spectral flow cytometer. Glucose-6-phosphate dehydrogenase (G6PD) enzymatic activity was explicitly measured from cellular lysates using a commercial Glucose-6-Phosphate Dehydrogenase Assay Kit (Sigma-Aldrich, Cat# MAK451-1KT) according to the manufacturer's instructions.

Machine Learning

A random forest classification model was trained in R (version 4.3.2) using the caret package to identify predictors of the binary response variable. Internal model tuning and validation were performed using repeated 20-fold cross-validation with five repeats. The random forest was fit with 1,000 trees, node size = 1, and permutation-based variable importance (nPerm = 10) using all available predictors. Model performance was summarized using a confusion matrix generated from model predictions, from which accuracy, sensitivity, specificity, positive predictive value (PPV), negative predictive value (NPV), F1 score, and balanced accuracy were calculated.

To evaluate discriminative performance, a logistic regression model was additionally fit using the same predictors, and receiver operating characteristic (ROC) curves with 95% confidence intervals were generated using the pROC package. The area under the curve (AUC) was used as the primary discrimination metric.

To assess the potential influence of clinical heterogeneity and test robustness of the predictive signal, we performed a sensitivity analysis comparing six model variants: full model, clinical variables only, lipid variables plus MMF dose, MMF dose only, lipid variables plus MMF dose plus statin use, and clinical variables plus lipid variables. These models were evaluated using the same random forest/logistic regression workflow and performance metrics, and results were compared across models to determine whether clinical covariates materially contributed to classification performance.

Tables

Table S1: Demographics of SOTR in observational study

Clinical characteristics of SOTRs identified as non-responders (NR) or responders (R) to a third COVID-19 vaccine dose determined by seroconversion of anti-S IgG on the MSD platform.

	Overall	NR	R	
n	44	13	31	P-value
**Demographic**				
Age (median [IQR])	64 [49, 69]	64.5 [48.7, 66.5]	64.0 [52.7, 69.9]	0.512
Sex, female (%)	23 (52.3)	8 (61.5)	15 (48.4)	0.641
Time (year) since Transplant (median [IQR])	4.48 [2.06, 10.88]	4.56 [2.07, 11.54]	4.39 [2.18, 10.57]	0.847
Race, n (%)				>0.99
White	42 (95)	12 (100)	30 (94)	
Asian	1 (2)	0 (0)	1 (3)	
Black	0 (0)	0 (0)	0 (0)	
Other	1 (2)	0 (0)	1 (3)	
**Transplant Organ**				
Kidney (%)	26 (59.1)	9 (69.2)	17 (54.8)	0.582
Liver (%)	10 (22.7)	1 (7.7)	9 (29)	0.251
Pancreas (%)	2 (4.5)	1 (7.7)	1 (3.2)	>0.99
Heart (%)	4 (9.1)	1 (7.7)	3 (9.7)	>0.99
Lung (%)	3 (6.8)	2 (15.4)	1 (3.2)	0.421
**Immunosuppressants**				
MMF/MPA daily dose (mg)	1000 [0, 1062.5]	1000 [1000, 1500]	0 [0, 1000]	0.005
MMF/MPA (%)	29 (65.9)	12 (92.3)	17 (54.8)	0.041
Azathioprine (%)	3 (6.8)	0 (0)	3 (9.7)	0.613
mTORi (evorilimus, sirolimus) (%)	7 (15.9)	2 (15.4)	5 (16.1)	>0.99
Corticosteroid (%)	24 (54.5)	10 (76.9)	14(45.2)	0.11
CNI (tacrolimus, cyclosporine) (%)	37 (84.1)	10 (76.9)	27 (87.1)	0.696
≥3 Medication (%)	17 (38.6)	9 (69.2)	8 (25.8)	0.018
**Primary vaccine types (%)**				0.308
Pfizer	13 (29.5)	4 (30.8)	9 (29)	
Moderna	17 (38.6)	3 (23.1)	14 (45.2)	
J&J	14 (31.8)	6 (46.2)	8 (25.8)	

Table S2: Demographics of SOTRs in CPAT Clinical Trial

Clinical characteristics of SOTRs identified as non-responders (NR) or responders (R) to a third COVID-19 vaccine dose determined by seroconversion of anti-S IgG on the MSD platform.

	Overall	NR	R	
n	31	4	27	P-value
**Demographic**				
Age (median [IQR])	67 [59, 75]	65 [55.5, 69.8]	70 [58.5, 74.5]	0.637
Sex, female (%)	11 (35.5)	1 (25)	10 (37)	>0.99
Time (year) since Transplant (median [IQR])	5.98 [2.84, 10.79]	9.59 [4.25, 15.49]	5.98 [2.82, 10.64]	0.48
Race, n (%)				>0.99
White	19 (61)	3 (75)	16 (59)	
Asian	1 (3)	0 (0)	1 (4)	
Black	8 (26)	1 (25)	7 (26)	
Other	3 (10)	0 (0)	3 (11)	
**Transplant Organ**				
Kidney (%)	31 (100.0)	4 (100.0)	27 (100.0)	--
Liver (%)	0 (0)	--	--	--
Pancreas (%)	0 (0)	--	--	--
Heart (%)	0 (0)	--	--	--
Lung (%)	0 (0)	--	--	--
**Immunosuppressants**				
MMF/MPA daily dose (mg)	750 [0,1000]	1500 [750, 2000]	500 [0, 1000]	0.136
MMF/MPA (%)	22 (71.0)	3 (75)	19 (70.4)	>0.99
Azathioprine (%)	0 (0)	--	--	
mTORi (evorilimus, sirolimus) (%)	0 (0)	--	--	
Corticosteroid (%)	28 (90.3)	4 (100)	24 (88.9)	>0.99
CNI (tacrolimus, cyclosporine) (%)	30 (96.8)	4 (100)	26 (96.3)	>0.99
≥3 Medication (%)	18 (58.1)	3 (75)	15 (55.6)	0.847
**Primary vaccine types (%)**				0.621
Pfizer	18 (58.1)	3 (75)	15 (55.6)	
Moderna	13 (41.9)	1 (25)	12 (44.4)	
J&J	0 (0)	0 (0)	0 (0)	

Table S3: Demographics of SOTRs in scRNA sequencing

Clinical characteristics of SOTRs identified as non-responders (NR) or responders (R) to a third COVID-19 vaccine dose determined by seroconversion of anti-S IgG on the MSD platform.

	Overall	NR	R
n	24	10	10
**Demographic**			
Age (median [IQR])	67.5 [57.5, 71.5]	66.0 [49.0, 72.0]	69.5 [63.0, 71.0]
Sex, female (%)	10 (50%)	6 (60%)	4 (40%)
Time (year) since Transplant (median [IQR])	3.1 [2.3, 9.2]	4.0 [2.2, 13.2]	3.0 [2.3, 7.6]
**Transplant Organ**			
Kidney (%)	11 (55%)	6 (60%)	5 (50%)
Liver (%)	5 (25%)	1 (10%)	4 (40%)
Pancreas (%)	0 (0)	--	--
Heart (%)	2 (10%)	1 (10%)	1 (10%)
Lung (%)	2 (10%)	2 (20%)	0 (0%)
**Immunosuppressants**			
Antimetabolites (%)	12 (60%)	8 (80%)	4 (40%)
Azathioprine (%)	0 (0)	--	--
mTORi (evorilimus, sirolimus) (%)	0 (0)	--	--
Corticosteroid (%)	9 (45%)	5 (50%)	4 (40%)
CNI (tacrolimus, cyclosporine) (%)	16 (80%)	9 (90%)	7 (70%)
**MMF Dose**			
High	9 (45%)	6 (60%)	3 (30%)
Low	5 (25%)	3 (30%)	2 (20%)
None	6 (30%)	1 (10%)	5 (50%)
**Primary vaccine types (%)**			
Pfizer	7 (35%)	4 (40%)	3 (30%)
Moderna	5 (25%)	1 (10%)	4 (40%)
J&J	8 (40%)	5 (50%)	3 (30%)

Table S4: Custom Lipid Related Gene sets

HALLMARK_CHOLESTEROL_HOMEOSTASIS

HALLMARK_FATTY_ACID_METABOLISM

REACTOME_ABC_TRANSPORTERS_IN_LIPID_HOMEOSTASIS

REACTOME_GLYCEROPHOSPHOLIPID_CATABOLISM

REACTOME_GLYCOSPHINGOLIPID_BIOSYNTHESIS

REACTOME_GLYCOSPHINGOLIPID_CATABOLISM

REACTOME_GLYCOSPHINGOLIPID_METABOLISM

REACTOME_GLYCOSPHINGOLIPID_TRANSPORT

REACTOME_REGULATION_OF_LIPID_METABOLISM_BY_PPARALPHA

REACTOME_LIPID_PARTICLE_ORGANIZATION

REACTOME_ROLE_OF_PHOSPHOLIPIDS_IN_PHAGOCYTOSIS

REACTOME_LYSOSPHINGOLIPID_AND_LPA_RECEPTORS

REACTOME_BIOSYNTHESIS_OF_THE_N_GLYCAN_PRECURSOR_DOLICHOL_LIPID_LINKED_OLIGOSACCHARIDE_LLO_AND_TRANSFER_TO_A_NASCENT_PROTEIN

REACTOME_METABOLISM_OF_LIPIDS

REACTOME_SPHINGOLIPID_CATABOLISM

REACTOME_SPHINGOLIPID_DE_NOVO_BIOSYNTHESIS

REACTOME_SPHINGOLIPID_METABOLISM

REACTOME_DIGESTION_OF_DIETARY_LIPID

REACTOME_PEROXISOMAL_LIPID_METABOLI

REACTOME_PHOSPHOLIPID_METABOLISM

REACTOME_GLYCEROPHOSPHOLIPID_BIOSYNTHESIS

REACTOME_CHOLESTEROL_BIOSYNTHESIS

REACTOME_NR1H2_NR1H3_REGULATE_GENE_EXPRESSION_TO_LIMIT_CHOLESTEROL_UPTAKE

REACTOME_NR1H3_NR1H2_REGULATE_GENE_EXPRESSION_LINKED_TO_CHOLESTEROL_TRANSPORT_AND_EFFLUX

REACTOME_REGULATION_OF_CHOLESTEROL_BIOSYNTHESIS_BY_SREBP_SREBF

REACTOME_BETA_OXIDATION_OF_VERY_LONG_CHAIN_FATTY_ACIDS

REACTOME_FATTY_ACID_METABOLISM

REACTOME_FATTY_ACIDS

REACTOME_FATTY_ACIDS_BOUND_TO_GPR40_FFAR1_REGULATE_INSULIN_SECRETIONREACTOME_FATTY_ACYL_COA_BIOSYNTHESIS

REACTOME_FREE_FATTY_ACID_RECEPTORS

REACTOME_FREE_FATTY_ACIDS_REGULATE_INSULIN_SECRETION

REACTOME_MITOCHONDRIAL_FATTY_ACID_BETA_OXIDATION

REACTOME_MITOCHONDRIAL_FATTY_ACID_BETA_OXIDATION_OF_SATURATED_FATTY_ACIDS

REACTOME_MITOCHONDRIAL_FATTY_ACID_BETA_OXIDATION_OF_UNSATURATED_FATTY_ACIDS

REACTOME_THE_FATTY_ACID_CYCLING_MODEL

REACTOME_TRANSPORT_OF_FATTY_ACIDS

Table S5: Antibody Staining Panel

Fluorophore	Specificity	Vendor	Clone	Catalog
**Surface**				
BB630	CD11c	BD-Crada	BU15	624294
BB660	FcRL5 (CD307e)	BD-Crada	509F6	624295
BB700	BTLA	BD	J168-540	746166
BB790	IgD	BD-Crada	IA6-2	624296
PE CF594	CD27	BD	M-T271	562297
PE Cy5	CD10	BD	HI10a	555376
PE Cy7	CXCR5	Biolegend	J252D4	356923
AF700	IgM	Biolegend	MHM88	314537
APC H7	CD19	BD	H1B19	560727
BUV395	CD24	BD	ML5	566221
BUV496	CD21	BD	B-ly4	624283
BUV661	CD38	BD	HIT2	565070
BUV737	IgG	BD	G18-145	612819
BUV805	CD22	BD	HIB22	742009
BV605	CD43	BD	1G10	563378
BV711	CD39	BD	Tu66	563680
BV750	FcyRII (CD32)	BD	3D3	747110
BV786	CD20	BD	TH7	743611
BV510	CD3	BD	UCHT1	563109
BV510	CD14	BD	MφP9	563079
Aqua	LD			
**Probes**				
Recombinant SARS-CoV-2 Spike-Prot (HEK)-Avitag		acrobiosystems	N/A	SPN-C82E9-200ug
BV650	Streptavidin	Biolegend	N/A	405229
FITC	Streptavidin	Biolegend	N/A	405201
PE	Streptavidin	Biolegend	N/A	100
**Intracellular**				
PECy5.5	CPT1a	Abcam	8F6AE9	ab128568
Dylight 680*	HK2	Abcam	EPR20839	ab228819
Alexa Fluor 532*	VDAC1	Abcam	20B12AF2	ab14734
Alexa Fluor 405	Tomm20	Abcam	20B12AF2	ab210047
Alexa Fluor 647	GLUT1	Abcam	EPR3915	ab195020

***Custom conjugated**

References

1. Biavati L, et al. An Allogeneic Multiple Myeloma GM-CSF–Secreting Vaccine with Lenalidomide Induces Long-term Immunity and Durable Clinical Responses in Patients in Near Complete Remission. *Clin Cancer Res*. 2021;27(24):6696–6708.

2. Nowicka M, et al. CyTOF workflow: differential discovery in high-throughput high-dimensional cytometry datasets [preprint]. 2019. https://doi.org/10.12688/f1000research.11622.3.

Supplemental Figure 1: Reduced class switched S-specific titers and B cells in SOTRs following two doses

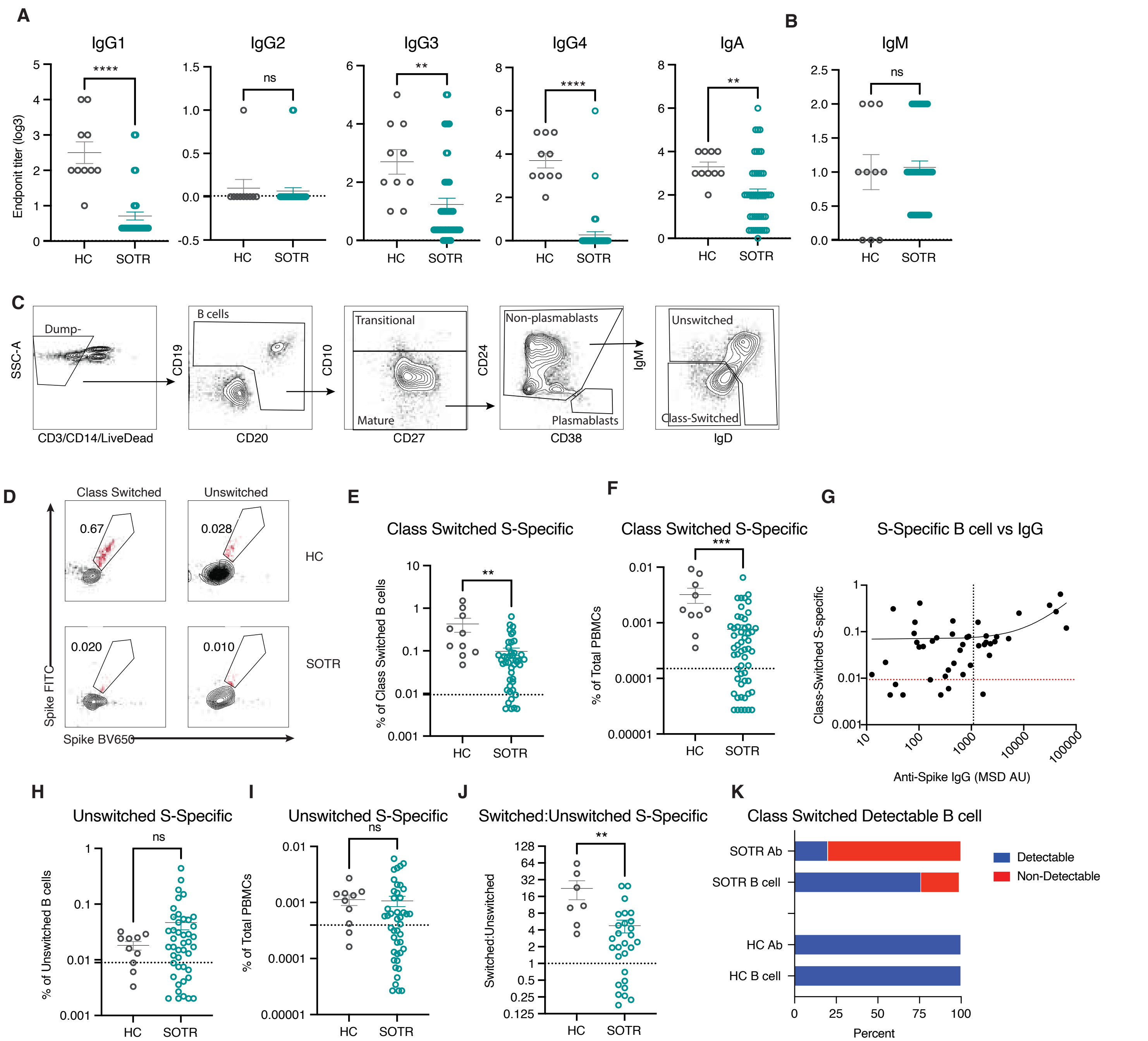

Supplemental Figure 1: Reduced class-switched S-specific titers and B cells in SOTRs following two vaccine doses.

Anti-S titers and S-specific B cells were evaluated in mRNA vaccinated HCs (n = 10) 6 months post dose two and SOTRs (n=44) 3-4 months post dose two, prior to dose three. A. Anti-S IgG subclasses and IgA titers. B. Anti-S IgM titers. C. Representative gating of basic B cell subsets. D. Representative staining of S-specific B cells. E-F. Frequency of class-switched S-specific B cells as frequency of total class-switched B cells (E) or total PBMCs (F). Detectable threshold denoted by dotted line. G. Correlation between anti-S IgG titers and frequency of S-specific B cells out of total class-switched B cells. H-I. Frequency of unswitched S-specific B cells as frequency of total unswitched B cells (H) or total PBMCs (I). J. Ratio of S-specific B cells with switched to unswitched phenotype. K. Proportion of participants with S-specific B cells or anti-S IgG titers above positive threshold. Data presented as Mean±SEM. Significance tested using Kruskall-Wallis test. *p < 0.05, **p < 0.01, ***p < 0.001, and ****p < 0.0001

Supplemental Figure 2: Third dose significantly increases anti-S titers and S-specific B cells in SOTRs

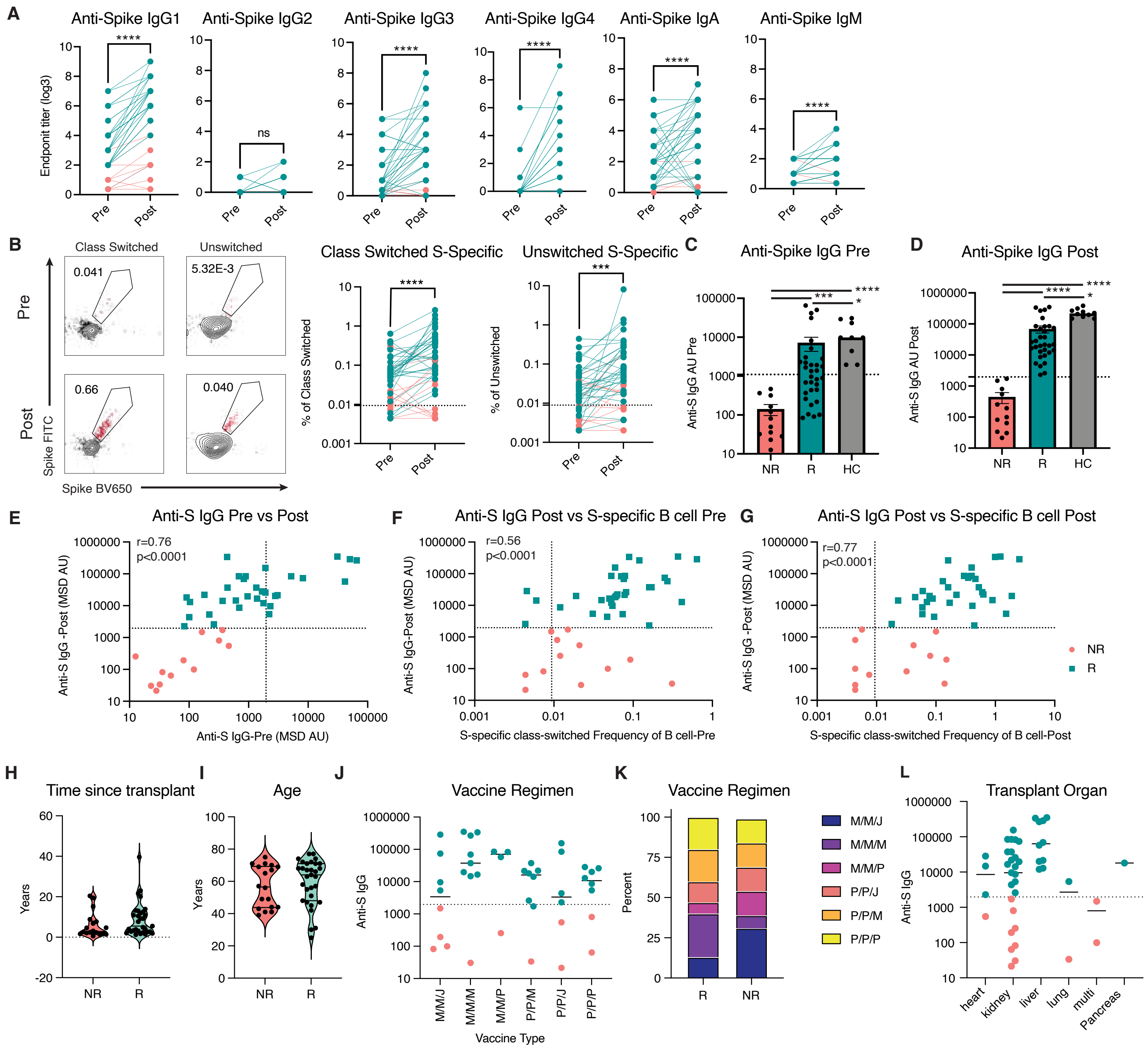

Supplemental Figure 2: Third vaccine dose significantly increases anti-S titers and S-specific B cell frequencies in SOTRS.
S-specific titers and anti-S B cells were evaluated in SOTRS (n=44) 3-4 months post dose two (Pre) and two weeks post dose three (Post). In all panels, responders (positive IgG titers after dose 3, green) and non-responders (pink) are indicated. A. Change in anti-S IgG subclasses, IgA, and IgM titers. B. Representative staining of S-specific B cells and frequency of class-switched or unswitched S-specific B cells. C. Anti-S IgG titers in non-responders (NR), responders (R) or healthy controls (HCs) pre-dose 3 D. Anti-S IgG titers in NR, R, or HCs at peak response post dose three (SOTRs) or dose two (HCs). E-G. Correlation between anti-S IgG titers and frequency of S-specific B cells as indicated. H-L Clinical features associated with responders (R) and non-responders (NR). H. Time since transplant (years). I. Age of participant (years). J. Anti-S IgG titers after dose three segregated by three dose vaccine regimens. Moderna (M), Pfizer (P), Johnson and Johnson (J). K. Vaccine regimen segregated by responders and non-responders. L. Anti-S IgG titers after dose three segregated by transplanted organ. Data presented as Mean±SEM. Significance tested using Kruskall-Wallis test. Correlation tested using Spearman correlation. *p < 0.05, **p < 0.01, ***p < 0.001, and ****p < 0.0001

Supplemental Figure 3: Variation in vaccine response on MMF not due to clinical features

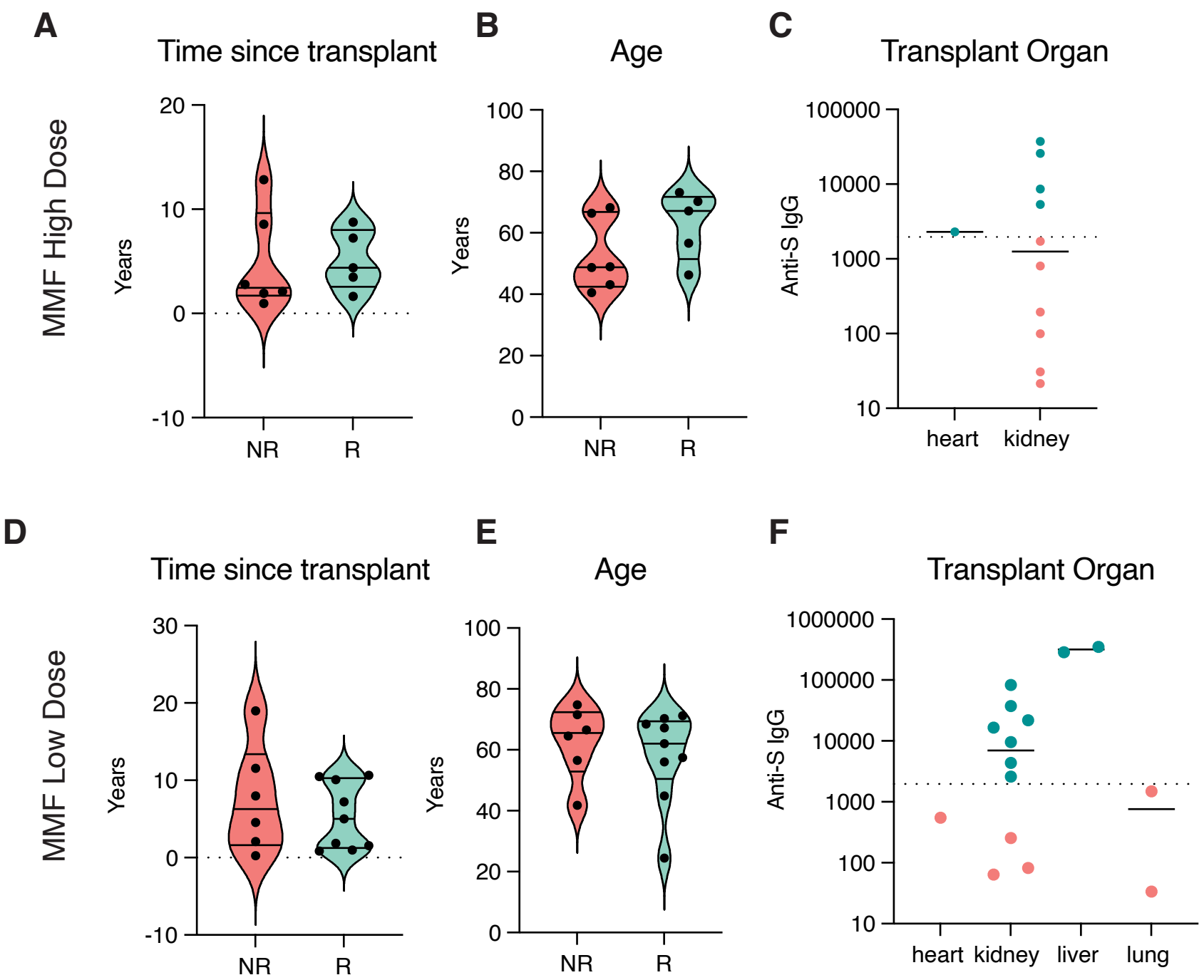

Supplemental Figure 3: Variation in vaccine response on MMF is not due to clinical factors
Clinical features associated with responders (R, pink) and non-responders (NR, green) taking high dose MMF/MPA (A-C) or low dose MMF/MPA (D-F). A,D. Time since transplant (years). B,E. Age of participant (years). C,F. Anti-S IgG titers after dose three segregated by transplanted organ. Significance tested using Mann-Whitney (A,B,D,E) or Kruskall-Wallis test (C,F).

Supplemental Figure 4: Immunosupressive drugs increase metabolic activity in immue cells

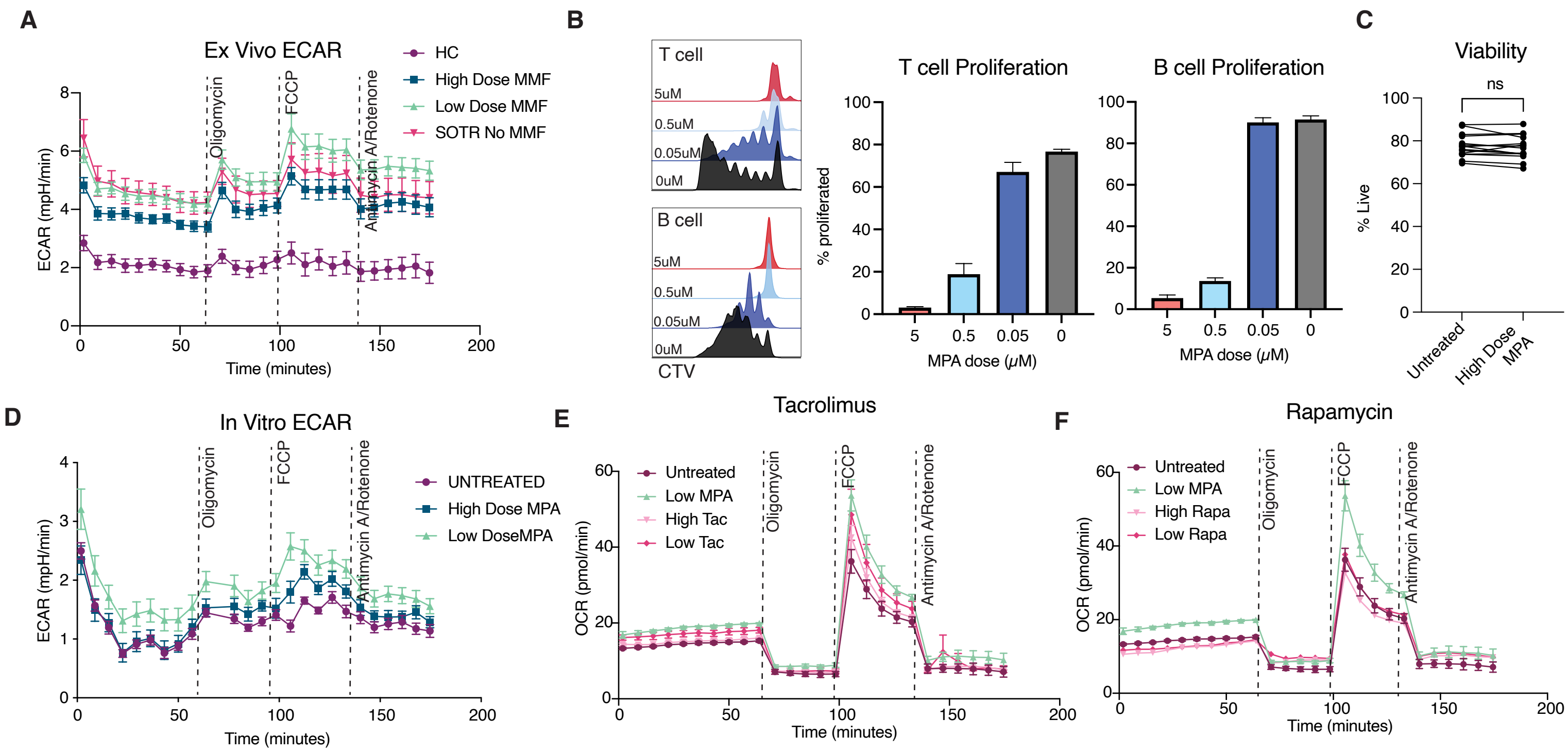

Supplemental Figure 4: Immunosupressive drugs increase metabolic activity in immune cells
A.Seahorse Extracellular Acidification Rate (ECAR) following mitochondrial stress test. ECAR of ex vivo PBMCs from SOTRs receiving no MMF, high dose MMF, or low dose MMF and HCs (n=3 per group and average of 4 replicates per condition) B. Cell Trace Violet staining of T cells stimulated with anti-CD3/anti-CD28 or B cells stimulated with CD40Ligand, CpG, and anti-IgG/IgM/IgA for 6 days in the presence of MPA at indicated dose. C. PBMCs were treated with high dose MPA (5μM) for 48 hours and viability was mesured by flow cytometry using Zombie NIR. D. ECAR of healthy PBMCs treated in vitro with high (5μM) or low dose (0.05μM) MPA for 48 hours followed by culture in limited nutrient media for 24 hours prior to mitochondrial stress test (n=3 per group and average of 4 wells per condition). E-F. OCR of healthy PBMCs treated in vitro with high dose Tacrolimus/FK506 (20ng/ml), low dose Tacrolimus/FK506 (0.2ng/ml), high dose Rapamycin (0.1μM) or low dose Rapamycin (0.001μM) as indicated for 24 hours followed by culture in limited nutrient media for 24 horus prior to mitochondrial stress test. Data presented as Mean±SEM.

Supplemental Figure 5: High dose MPA inducess mitochodndrial unresponsiveness

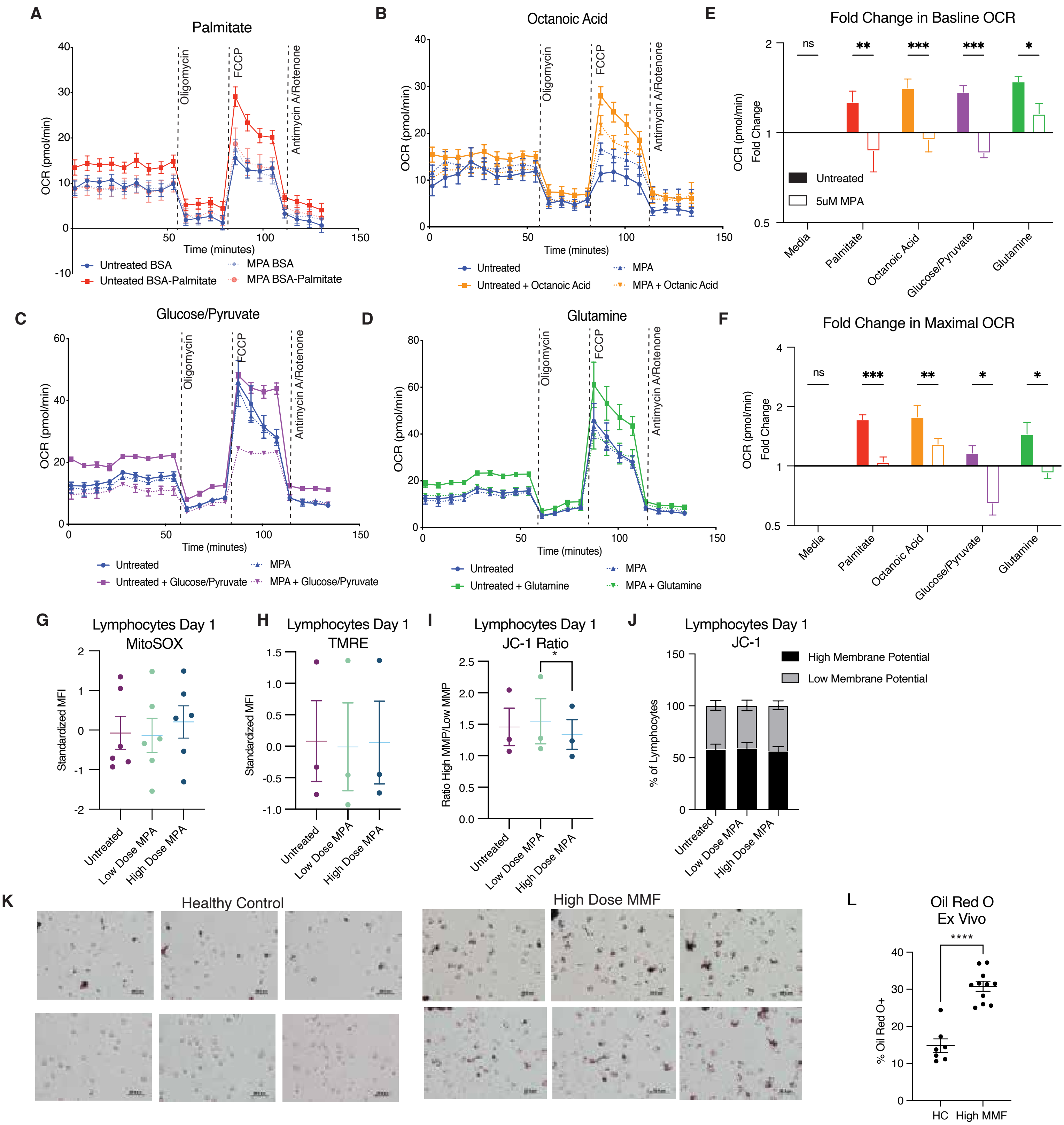

Supplemental Figure 5: High dose MPA induces mitochondrial unresponsiveness

A-D. Healthy PBMCs were treated with 5μM MPA for 24 hours in complete media then rested overnight in low-nutrient media prior to analysis. Oxygen consumption rate (OCR) of PBMCs exposed to long chain fatty acid palmitate (165 μM) (A), medium chain fatty acid octanoic acid (200μM) (B), glucose (10mM) and pyruvate (1mM) (C), or glutamine (2mM) (D) 20 minutes prior to analysis. E-F. Fold change in baseline (E) or maximal (F) OCR when healthy PBMCs were supplied with indicated substrates. G-I PBMCs were treated with high (5μM) or low dose (0.05μM) MPA for 24 hours and evaluated for mitochondrial health using by measuring reactive oxygen species (ROS; MitoSOX) (G), and mitochondrial membrane potential by uptake of tetramethylrhodamine ethyl ester (TMRE) (H) and JC-1 aggregation (I-J). MFI was standardized by experimental batch. K-L. PBMCs were stained with Oil Red O directly post thaw to measure lipid droplet accumulation. I. The proportion of cells occupied by Oil Red O staining was quantified using Cell Profiler, each dot represents one field of vision. Data presented as Mean±SEM. Significance tested using Kruskall-Wallis test. *$p < 0.05$, **$p < 0.01$, ***$p < 0.001$, and ****$p < 0.0001$

Supplemental Figure 6: Depletion of guanosine nucleotides leads to lipid accumulation in multiple immune cell subsets

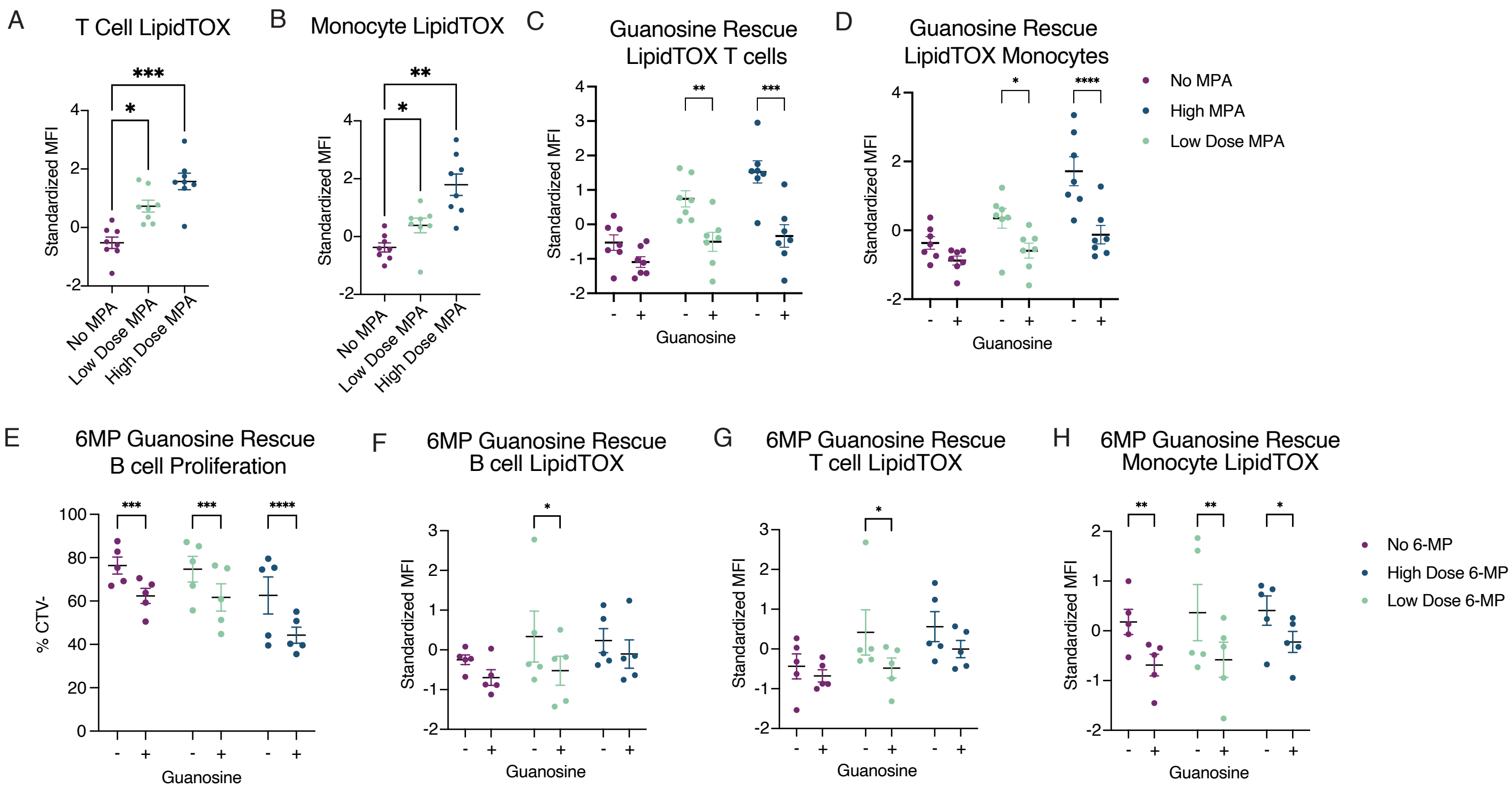

Supplemental Figure 6: Depletion of guanosine nucleotides leads to lipid accumulation in multiple immune cell subsets

A-B. PBMCs were stimulated with CpG (2.5μg/mL) for 6 days in the presence of high (5μM) or low dose (0.05μM) MPA. T cells (A) and monocytes (B) were evaluated for total lipid content using lipidTOX staining. Mean Fluorescent Intensity (MFI) was standardized by experimental batch. Significance tested using Friedman test. C-D. PBMCs were stimulated as described in A-B and supplemented with guanosine (25 μM). T cells (C) and monocytes (D) were evaluated for lipid accumulation by lipidTOX staining. E-H. PBMCs were stimulated with CpG (2.5μg/mL) for 6 days in the presence of high (5μM) or low dose (0.05μM) 6-MP and supplemented with guanosine (25 μM). B cells were evaluated for proliferation as determined by %cell trace violet low (CTV-) (E) and immune cell subsets were evaluated for lipid accumulation by lipidTOX staining (F-H). Mean Fluorescent Intensi.vvty (MFI) was standardized by experimental batch. Data presented as Mean±SEM. Significance tested using 2-way ANOVA unless otherwise noted. $^*p < 0.05$, $^{**}p < 0.01$, $^{***}p < 0.001$, and $^{****}p < 0.0001$.

Supplemental Figure 7: CD11c+ B cells representative gating

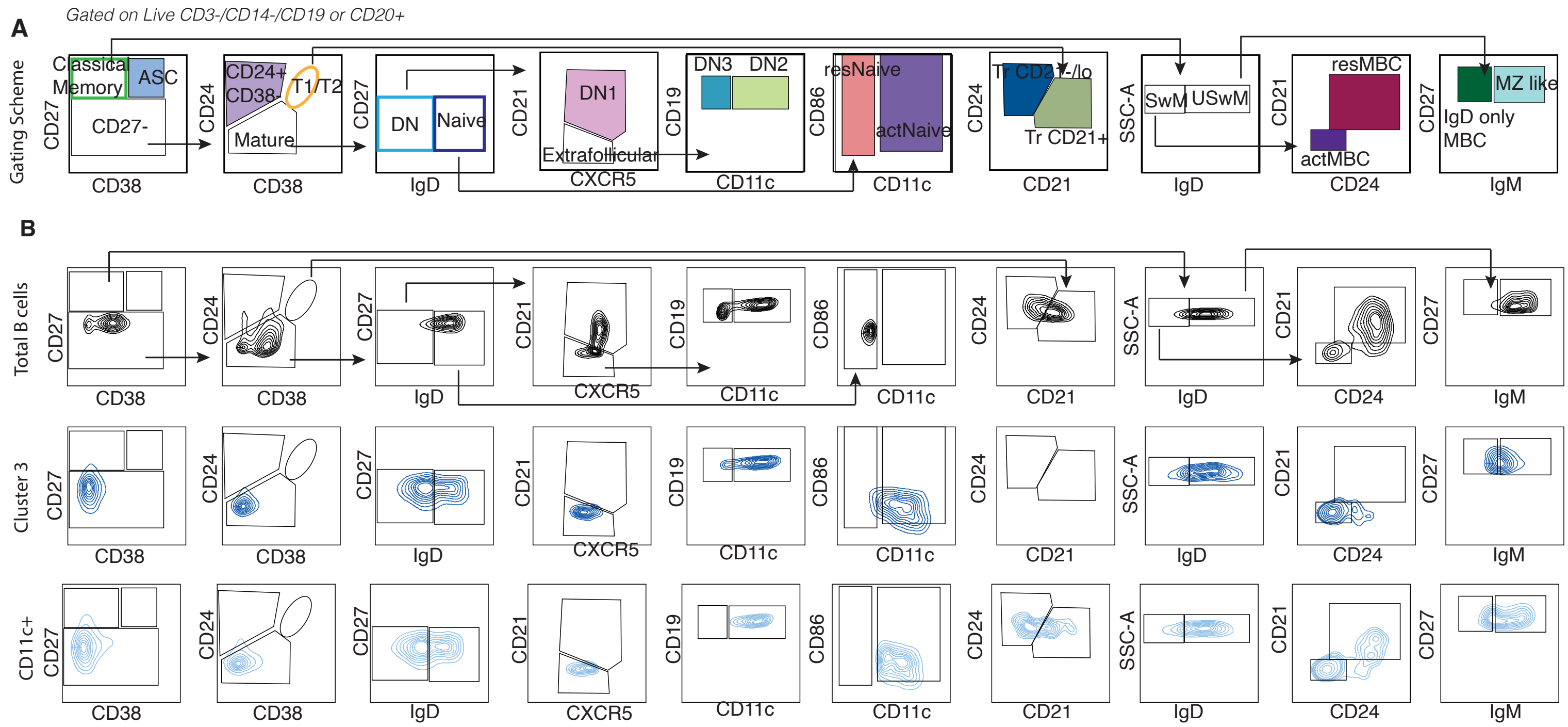

Supplemental Figure 7: CD11c+ B cell representative gating

A.Gating scheme used to identify canonical B cell subsets. Gates are colored in accordance with pie charts in Figure 3F. B Representative gating shown for concatenated file of total B cells, cluster 3 B cells, and CD11c+ B cells.

Supplemental Figure 8: CD11c+ B cells support vaccine response

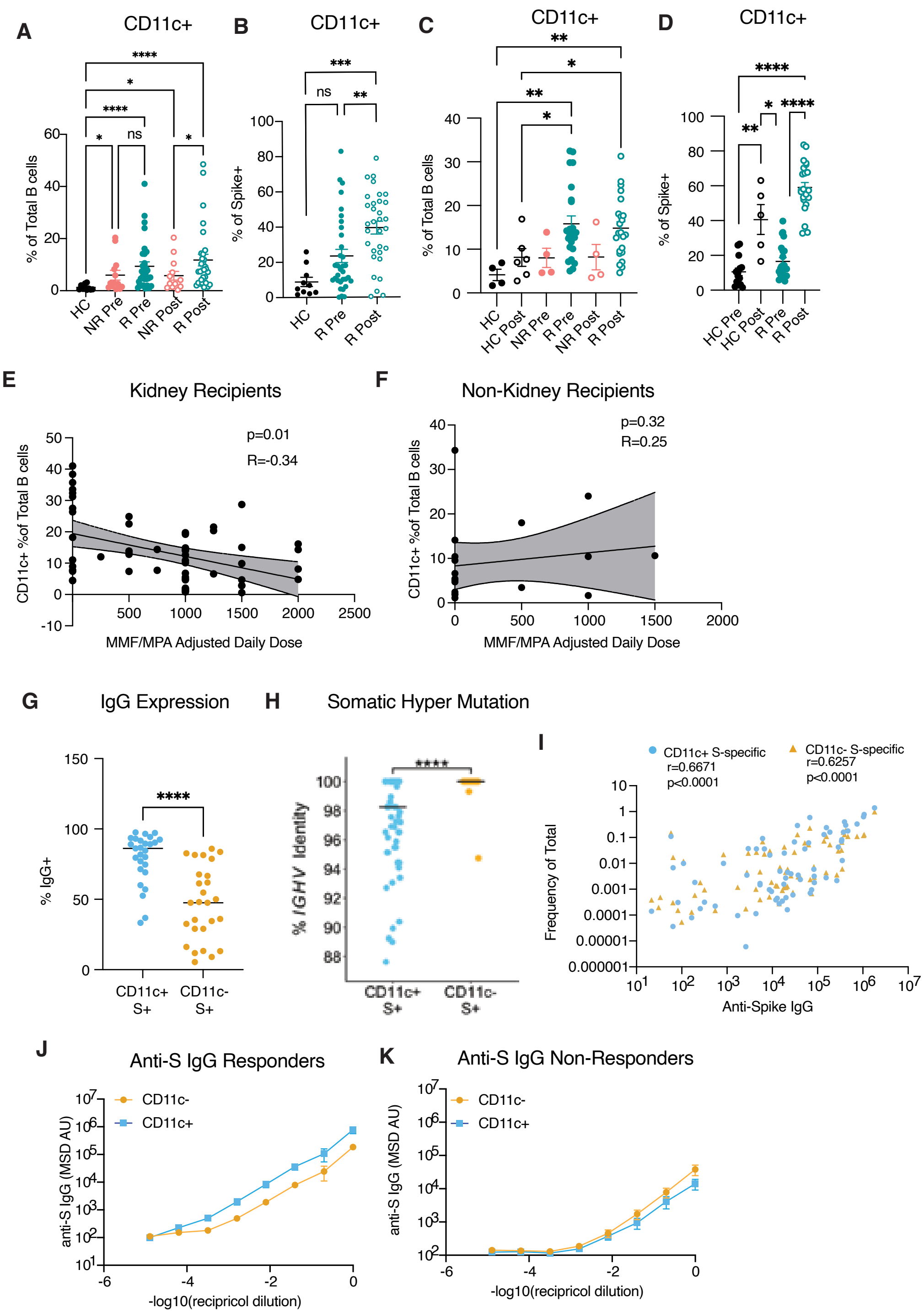

Supplemental Figure 8: CD11c+ B cells support vaccine response
A-B. Frequency of CD11c+ B cells out of total or spike (S)-specific B cells for SOTRs in observational cohort pre-dose 3 and two weeks post-dose 3. C-D. Frequency of CD11c+ B cells out of total or S-specific B cells for SOTRS in clinical trial NCT04969263 pre-dose 3 and two weeks post-dose 3. E-F. Frequency of CD11c+ B cells out of total B cells measured pre-vaccination correlated with adjust daily MMF/MPA dose in kidney transplant recipients (E) or recipients of non-kidney organs (F). G. Proportion of CD11c+ or CD11c- S-specific B cells from SOTRs that express IgG within NCT04969363 cohort as assesed by flow cytometry. H. Somatic mutation of the IGHV gene expressed as percent identity to germline IGHV for CD11c positive and negative S-specific B cells. I. Correlation between CD11c+ S-specific or CD11c- S-specific B cells and anti-S IgG titers. Data from combined cohorts. Correlation tested using Spearman correlation. J-K. CD11c+ or CD11c- B cells were sorted from SOTR responders (J) or non-responders (K) and stimulated with CpG, CD40L, and IL-21 for 6 days. On day 6 supernatants were collected and assayed for secretion of anti-S IgG Abs. Data presented as Mean±SEM. Significance tested using Kruskall-Wallis test. Correlation tested using Spearman correlation. *p < 0.05, **p < 0.01, ***p < 0.001, and ****p < 0.0001

Supplemental Figure 9: MPA induced lipid oxidation is associated with CPT1A expression

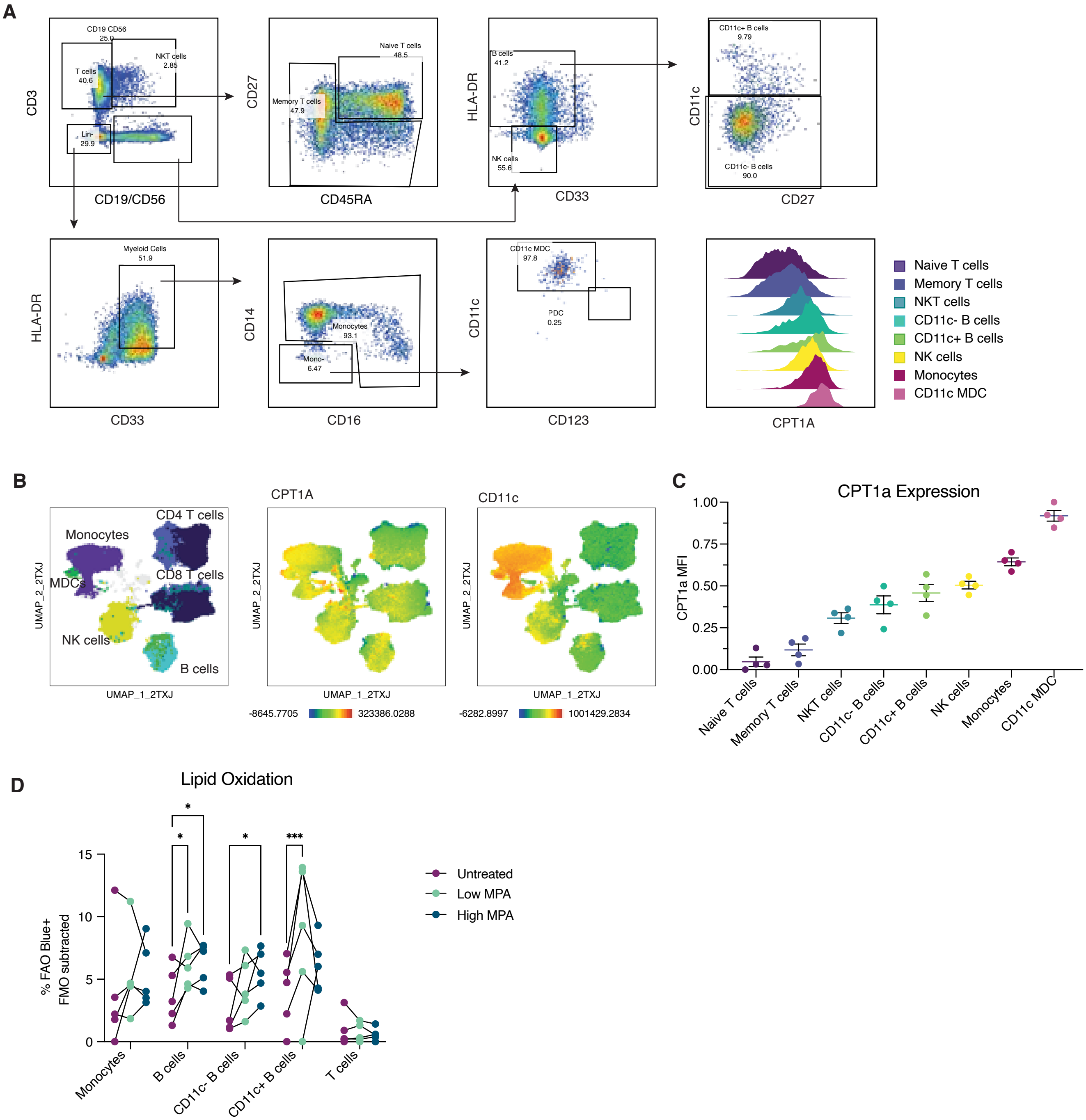

Supplemental Figure 9: MPA induced lipid oxidation is associated with CPT1A expression
A-C.Healthy PBMCs were evaluated for CPT1A expression by flow cytometry. A, Representative gating of PBMC subsets and overlaid histogram of CPT1A expression. B. UMAP of total PBMC populations with major cell subsets identified and CPT1A and CD11c expression shown as heatmap. C. Quantification of normalized CPT1A mean fluorescence intensity (MFI) across immune cell subsets. D. Healthy PBMCs were treated in vitro with high (5μM) or low dose (0.05μM) MPA for 24 hours followed by culture in limited nutrient media for 24 hours prior to analysis with FAO-blue. Frequencies of FAO-blue positive cells in the Fluorescence minus one (FMO) controls were subtracted for each cell type by donor. Significance tested using 2-way ANOVA otherwise stated. *$p < 0.05$, **$p < 0.01$, ***$p < 0.001$, and ****$p < 0.0001$

Supplemental Figure 10: MPA induces lipid accumulation through synthesis not uptake in B cells

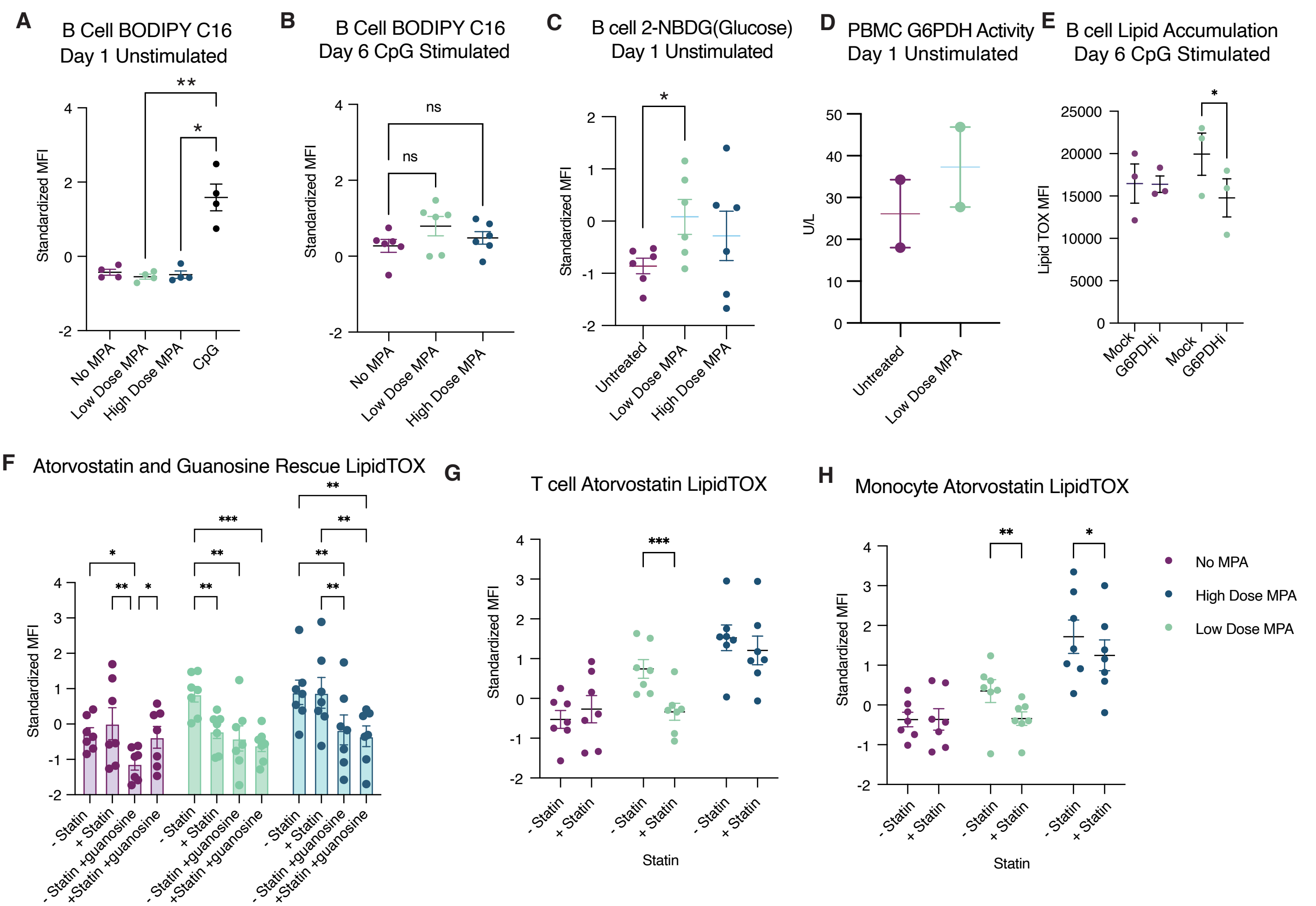

Supplemental Figure 10: MPA induces lipid accumulation through synthesis not uptake in B cells
A.Healthy PBMCs were treated in vitro with high (5μM), low dose (0.05μM) MPA , or CpG (2.5μg/mL) overnight. Cells were then treated with fluorescently labeled long chain fatty acid (BODIPY C16) and stained by flow cytometry to evaluate B cell lipid uptake. B.Healthy PBMCs were treated in vitro with high (5μM), low dose (0.05μM) MPA in the presence of CpG (2.5μg/mL) for 6 days. On day 6 cells were then treated with fluorescently labeled long chain fatty acid (BODIPY C16) and stained by flow cytometry to evaluate B cell lipid uptake. Significance tested using Friedman test. D. Healthy PBMCs were treated in vitro with high (5μM), low dose (0.05μM) MPA overnight and assayed for G6PDH activity. E.Healthy PBMCs were stimulated with CpG (2.5μg/mL) for 6 days in the presence of high (5μM) or low dose (0.05μM) MPA. Cells were simultaneously supplemented with G6PDH inhibitor (G6PDHi, 10μM). On day 6, total B cells were evaluated for lipid droplet accumulation as assessed by lipidTOX staining. F-H. Healthy PBMCs were stimulated with CpG (2.5μg/mL) for 6 days in the presence of high (5μM) or low dose (0.05μM) MPA. Cells were simultaneously supplemented with atorvastatin (HMGCoA inhibitor, 0.02μM), and/or guanosine (25 μM). On day 6, total B cells were evaluated for lipid droplet accumulation as assessed by lipidTOX staining. Data presented as Mean±SEM. Significance tested using 2-way ANOVA unless otherwise stated. *$p < 0.05$, **$p < 0.01$, ***$p < 0.001$, and ****$p < 0.0001$

Supplemental Figure 11: Measurements of lipid metabolism improve vaccine response prediction

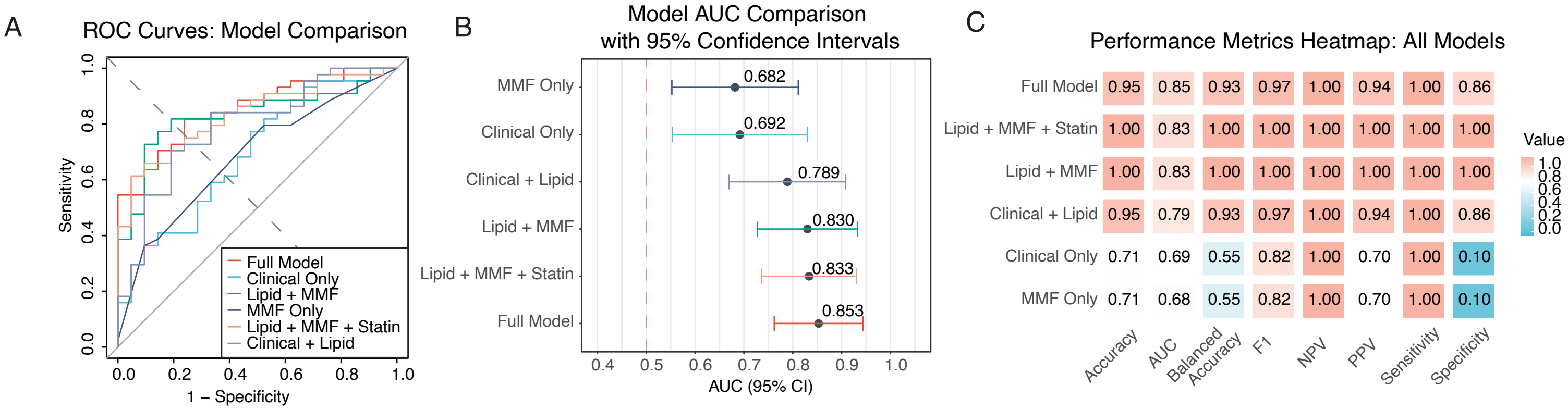

Supplemental Figure 11: Measurements of lipid metabolism improve vaccine response prediction
(A) Receiver operating characteristic (ROC) curves comparing the full model and reduced models. (B) Area under the curve (AUC) estimates with 95% confidence intervals for each model. (C) Heatmap summarizing model performance metrics, including accuracy, AUC, balanced accuracy, F1 score, positive predictive value (PPV), negative predictive value (NPV), sensitivity, and specificity. The full model and the lipid + MMF model showed the strongest overall performance, whereas clinical-only and MMF-only models performed poorly.
